# Early Diagnosis and Management of Nitrogen Deficiency in Plants Utilizing Raman Spectroscopy

**DOI:** 10.3389/fpls.2020.00663

**Published:** 2020-06-05

**Authors:** Chung Hao Huang, Gajendra Pratap Singh, Su Hyun Park, Nam-Hai Chua, Rajeev J. Ram, Bong Soo Park

**Affiliations:** ^1^Temasek Life Sciences Laboratory, National University of Singapore, Singapore, Singapore; ^2^Disruptive & Sustainable Technologies for Agricultural Precision, Singapore-MIT Alliance for Research and Technology, Singapore, Singapore; ^3^Research Laboratory of Electronics, Massachusetts Institute of Technology, Cambridge, MA, United States

**Keywords:** nitrogen deficiency, Raman spectroscopy, nitrate peak, Arabidopsis, leafy vegetables

## Abstract

Nutrient deficiency alters growth and development of crop plants and compromises yield. Real-time non-invasive monitoring of the nutritional status of crops would allow timely applications of fertilizers to optimize for growth and yield at different times of the plant’s life cycle. Here, we used Raman spectroscopy to characterize Arabidopsis and two varieties of leafy vegetable crops under nitrogen sufficient and deficient conditions. We showed that the 1046 cm^–1^ Raman peak serves as a specific signature of nitrogen status *in planta*, which can be used for early diagnosis of nitrogen deficiency in plants before onset of any visible symptoms. Our research can be applied toward crop management for sustainable and precision agriculture.

## Introduction

Precision farming deploys intelligent systems to increase agricultural productivity and profitability while protecting the environment. Sensors can play a valuable role in providing timely, spatially-resolved measurements of biophysical parameters that can guide management decisions. For example, fertilizer application can be tailored to specific crop health. Nitrogen is generally the most important and also the major limiting factor for crop growth and agriculture productivity (Masclaux-Daubresse et al., 2010; Kant et al., 2011). Nitrogen concentration in plant vegetation is related to chlorophyll content and photosynthesis efficiency. Nitrogen-limiting conditions promote leaf senescence lowering yield and biomass in plants (Kant et al., 2011). However, when nitrogen supply surpasses vegetation’s nutritional needs, the excess is eliminated by runoff and infiltration into the water table leading to pollution of aquatic ecosystems resulting in eutrophication. Further environmental pollution is linked to the production of nitrous oxides and the fossil fuels consumed in the production of ammonia (Santamaria, 2006; Ju et al., 2009). Precision agriculture seeks to limit this pollution by using sensor data to deliver precisely enough fertilizer to meet the nutritional needs of plants.

Previous work on optical sensing of nitrogen deficiency has relied on measuring the effect of nutrient stress on chlorophyll content, foliage reflectance and transmittance, via a reduction in chlorophyll, which were found to be affected by nitrogen deficiency. However, changes in the spectral reflectance due to nitrogen deficiency have been shown to overlap with the spectral response due to other nutrient deficient stresses (Chappelle et al., 1992; Peñuelas et al., 1994; Blackmer et al., 1996) and to general stress response (Carter, 1994; Altangerel et al., 2017; Farber and Kurouski, 2018; Sanchez et al., 2019).

Here, we report on the specific, early detection of nitrogen status in plants using Raman spectroscopy. Raman spectroscopy, discovered in Raman and Krishnan (1928), measures the inelastic scattering of laser light that results in a characteristic “fingerprint” of vibrational frequencies for various molecular species present in a sample. Early experiments on aqueous salts of nitrate established the strong Raman peaks near 1049 cm^–1^ associated with the symmetric stretching of the three oxygen atoms of the nitrate ion (Grassmann, 1932; Silveira and Bauer, 1932). Here, we demonstrate that this nitrate Raman peak can be measured non-invasively in leaves and serves as an early and specific indicator for nitrogen status in plants.

## Materials and Methods

### Plant Materials, Growth Conditions and Preparation of Plant Samples

*Arabidopsis thaliana* WT (Col-0) and two leafy vegetables, Pak Choi (*Brassica rapa chinensis*) and Choy Sum (*Brassica rapa var. parachinensis*), were used. The *nrt2.1-2* mutant in the Col-0 background was obtained from the Salk Institute (*Salk_035429*). Seeds were germinated on 0.8% agar media containing Murashige and Skoog (MS) salts, 0.5 g/L MES and 10 g/L sucrose. Arabidopsis and vegetables were grown at 22°C with 60% relative humidity in long-day conditions (16 h light/8 h dark) under white light at 100 μmol m^–2^ s^–1^ in a growth chamber. Plants were grown in either +N or −N medium by modified Hoagland’s solution containing 2 mM CaCl_2_ and 3 mM KCl (pH 5.8) instead of 2 mM Ca(NO_3_)_2_ and 3 mM KNO_3_ (pH 5.8). After 3 days leaf yellowing which reflects nitrogen deficiency was not yet seen in plants grown on -N medium. For phosphate or potassium deficiency, we have replaced KH_2_PO_4_ with KCl, or KNO_3_ and KH_2_PO_4_ with NaNO_3_ and NaH_2_PO_4_, respectively. The number of biologically independent repeats in each experiment was described in figure legends.

### *nrt2.1-2* Mutant Genotyping

Arabidopsis genotypes were analyzed by Phire Plant Direct PCR Kit (Thermo Scientific). Briefly, 10 mg leaf sample was ground into a powder and dissolved in 10 ul dilution buffer. Total DNA extract was analyzed by PCR with gene-specific primer sets (Supplementary Table S1).

### Total Chlorophyll Content Measurement

Arabidopsis (Col-0 and *nrt2.1-2*) and two leafy vegetables (Pak Choi and Choy Sum) grown for 3 and 5 days, respectively, on +N or −N medium were used for total chlorophyll measurement. Leaves were extracted with 80% acetone at 4°C for 24 h in darkness. Total chlorophyll per fresh weight of leaf No. 4 samples was calculated as described previously (Porra et al., 1989). The number of biologically independent repeats in each experiment was described in figure legends.

### Nitrate Content Measurements

Nitrate content was determined as described previously (Cataldo et al., 1975). Briefly,100 mg leaf tissue was homogenized in 1 mL deionized water and incubated at 100°C for 20 min. 10 μL of the supernatant was mixed with 40 ul 5%(w/v) salicylic-sulphuric acid and the mixture incubated at room temperature for 20 min. Following addition of 950 μL 8% NaOH, the mixture was placed at room temperature for 20 min before O.D. at 410 nm was measured. The number of biologically independent repeats in each experiment was described in figure legends.

### RNA Extraction and Quantitative RT-PCR Analysis

Total RNA was isolated from Arabidopsis (Col-0) and the two leafy vegetables (Pak Choi and Choy Sum) using QIAGEN RNeasy Mini Kits (QIAGEN) according to the manufacturer’s instructions. Reverse transcriptional reaction was performed using iScript^TM^ cDNA Synthesis Kit (Bio-Rad) following to the manufacturer’s instructions. Quantitative RT-PCR was performed using the CFS96 real-time system (Bio-Rad) with *ORE1*, *NRT2.1*, *NRT2.2* specific primers and *ACT2* as a reference gene, or *ORE1* and *ACT2* orthologous gene for two leafy vegetables (Supplementary Table S1). The number of biologically independent repeats in each experiment was described in figure legends.

### Raman Methods

Raman spectra were collected using a purpose-built system designed for 830 nm excitation. The sample holder featured a 100 μm thick fused silica sampling window used for both excitation and collection of the Raman signal. An aspheric lens was used to focus the excitation light and collect the Raman scattered light. The lens was chosen with a depth of focus >1 mm so that Raman signal from the entire cross-section of a leaf was collected. The excitation laser used with this system was a fiber coupled laser (Innovative Photonic Solutions, United States) operating at 830 nm delivering approximately 100 mW of laser power to the sample. Light was delivered from the laser to collimating optics via a 105-micron core multimode fiber. The collimated light was passed through a Semrock MaxLine Laser Line 830 filter (Semrock Inc., United States) to remove any amplified spontaneous emission from the laser and any background generated within the delivery fiber. The filtered light was coupled into the optical path of the excitation lens by a Semrock long pass filter (Semrock Inc., United States) operated as a dichroic mirror. Collected light was passed back through the Semrock filter and then through an additional long pass filter to further attenuate Rayleigh scattered excitation light before being delivered to the spectrometer using an F# matching lens. Spectra were acquired using Kymera 328i spectrograph (Andor, United Kingdom) employing a 600 g/mm optical grating. For each sample of plant leaf, 5 spectra were collected with an integration time of 10 s per sample spot. Cosmic ray events were identified in the 10 s spectra and removed. After cosmic ray removal, the individual 10 s spectra were smoothed across wavelength using the Savitzky-Golay filter function (MATLAB Inc., United States) with a degree of 11. A representative sample spectrum was created by taking the mean value of the five filtered and smoothed spectra at each wavelength. The sample spectrum resulting from this processing contained Raman and fluorescence signal primarily from the leaf. To generate the leaf Raman spectra presented in the results section any residual fluorescence was removed by performing a positive residual style polynomial subtraction as described in reference (Lieber and Mahadevan-Jansen, 2003). Calibration of the Raman shift was performed using a polystyrene sample with a well-known Raman spectrum (Creely et al., 2005). The number of biologically independent repeats in each experiment was described in figure legends.

### Principal Component Analysis (PCA)

The spectra were analyzed in the Raman shift wavenumber range of 900–1600 cm^–1^ across 5 locations across 3 biological replicates for plants and leaves of the same age grown under +N and −N conditions. The eigenvectors of the covariance matrix of the original data set define the principal components (PCs) - the maximal directions of variance within a dataset.

## Results

**FIGURE 1:**
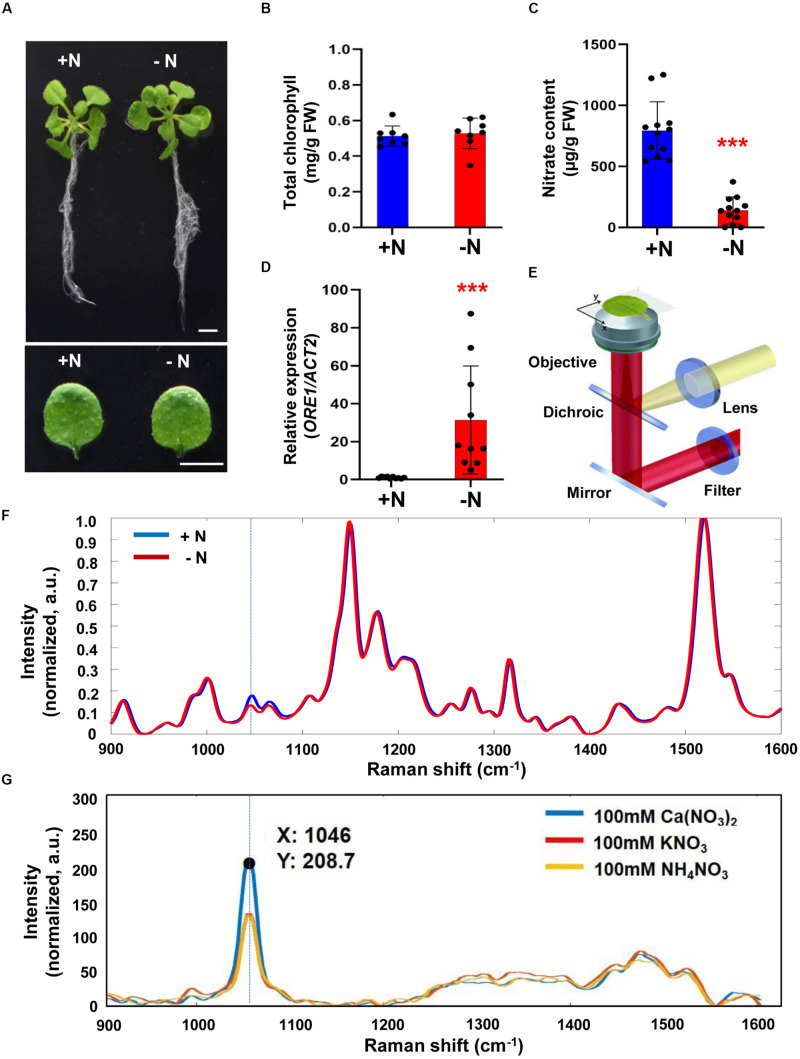
Analysis of biological/molecular phenotype and Raman spectrum of early nitrogen deficiency in Arabidopsis. Three-week-old seedlings of wild-type (WT) Arabidopsis (Col-0) were transferred into nitrogen-sufficient (+N) or nitrogen-deficient (–N) hydroponic medium and grown for an additional 3 days. **(A)** Morphological phenotype, scale bar, 1 cm. **(B)** Total chlorophyll content of leaf No. 4 was analyzed in +N and –N plants. *n* = 8 (biologically independent experiments). Data are mean values, *n* = 8 (biologically independent experiments) and individual data points are shown as overlays. FW; fresh weight. *P* values are given in Supplementary Table S2. **(C)** Nitrate content of leaf No. 4 was analyzed in +N or –N plants. Data are mean values, *n* = 12 (biologically independent experiments) and individual data points are shown as overlays. Asterisks indicate statistically significant difference compared with + N. *P < 0.05, **P < 0.01, and ***P < 0.001; two-tailed *t*-test. FW; fresh weight. Supplementary Table S2 shows *P* values. **(D)** *ORE1* transcript levels were analyzed by qRT-PCR in leaf No.4 samples of +N or -N plants. Data are mean values, *n* = 10 (biologically independent experiments) and individual data points are shown as overlays. Asterisks indicate statistically significant difference compared with + N. *P < 0.05, **P < 0.01, and ***P < 0.001; two-tailed *t*-test. Supplementary Tables S1, S2 show primer sets and *P* values, respectively. **(E)**. Schematic of the Raman spectroscopy setup. **(F)**. Three-week-old seedlings of WT transferred into +N and –N hydroponic medium and grown for 3 days. Leaf No. 4 was used for measurement of Raman spectra. Peak intensities are mean values, *n* = 12 (biologically independent experiments). **(G)**. Raman spectra of 100 mM Ca(NO_3_)_2_, KNO_3_, and NH_4_NO_3_. Identification of the nitrate peak by standard pure chemicals. a.u; arbitrary unit.

We investigated possible correlation between intensity changes of Raman spectra and nitrogen status in the model plant *Arabidopsis thaliana* where metabolic pathways are well studied and mutants affected in specific metabolic pathways are available. Three-week-old Arabidopsis plants were grown under sufficient (+N; complete) or nitrogen-deficient (−N) hydroponic media. A typically visible phenotype of nitrogen deficiency is the degradation of chlorophyll leading to leaf yellowing. However, no visible difference in leaf color was seen between plants grown for 3 days under the two conditions (+N and -N) (Figure 1A) and no measurable difference in the leaf chlorophyll content was detected (Figure 1B). However, despite the similarity in visible plant phenotype and chlorophyll content, chemical analysis showed the nitrate content of −N plants was decreased by 8-fold, compared to +N plants (Figure 1C). Moreover, plants grown under −N conditions were indeed experiencing stress responses because transcript levels of *ORE1*, a nitrogen-starvation induced gene (Park et al., 2018), were 30-fold higher in plants grown under −N conditions compared to +N plants (Figure 1D). These results establish that plants mount a response to nitrogen availability within short time-periods even when visible phenotypic changes associated with nitrogen-deficient stress had not yet appeared.

We wanted to explore if Raman spectroscopy can be used for early diagnosis of nitrogen deficiency in plants. Figure 1E shows a proposed Raman spectroscopy design for plant leaf analysis. The entire functioning components of Raman spectroscopy that were used in this work are shown in Supplementary Figure S1.

We compared Raman spectra of leaves from +N and −N plants and found differences in the intensity of Raman shifts at 1000 to 1100 cm^–1^ (Figure 1F). We measured Raman spectra of calcium nitrate [Ca(NO_3_)_2_], potassium nitrate (KNO_3_) and ammonium nitrate (NH_4_NO_3_) and all 3 compounds showed a peak at 1046 cm^–1^ indicating that this Raman shift (associated with the symmetrical stretching of nitrate) is indeed the nitrate peak (Figure 1G).

**FIGURE 2:**
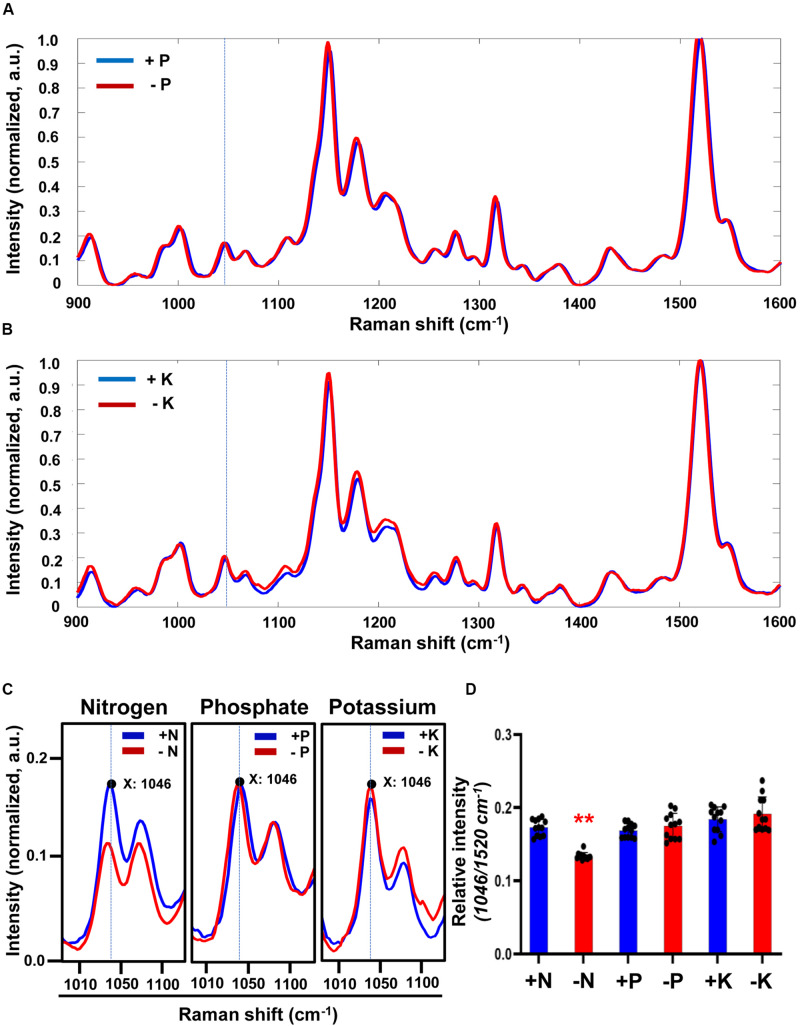
Comparison of Raman spectra under sufficient or deficient condition of 3 macronutrients (N, P, and K) in Arabidopsis. **(A,B)** Three-week-old seedlings of WT transferred into phosphate-sufficient (+P), phosphate-deficient (–P), potassium-sufficient (+K), or potassium-deficient (–K) hydroponic medium and grown for 3 days. Leaf No. 4 was used for measurement of Raman spectra. Peak intensities are mean values, *n* = 12 (biologically independent experiments). **(C)** Comparison of peak intensity of the 1046 cm^–1^ peak in +N, –N, +P, –P, +K or –K plants. Region of Raman spectra between 1010 and 1100 cm^–1^ is shown from Figure 1E, 2A,B. a.u; arbitrary unit. **(D)** The intensity of the 1046 cm^–1^ peak was analyzed. Data are mean values, *n* = 12 (biologically independent experiments) and individual data points are shown as overlays. Asterisks indicate statistically significant difference compared with +N, +P or +K, respectively. *P < 0.05, **P < 0.01, and ***P < 0.001; two-tailed *t*-test. *P* values are shown in Supplementary Table S3.

Three macronutrients are required for plant growth and development: nitrogen (N), phosphate (P) and potassium (K). To confirm the specific association of the 1046 cm^–1^ peak with nitrate deficiency we determined Raman spectra of plants starved with P or K (Figures 2A,B). Although there were changes between the Raman spectra of +P and −P plants no significant difference in the peak intensity at 1046 cm^–1^ was detected. Similar results were found for +K or −K plants (Figures 2C,D). These results show that the Raman peak at 1046 cm^–1^ can be used as a specific signature for plants grown under −N conditions.

**FIGURE 3:**
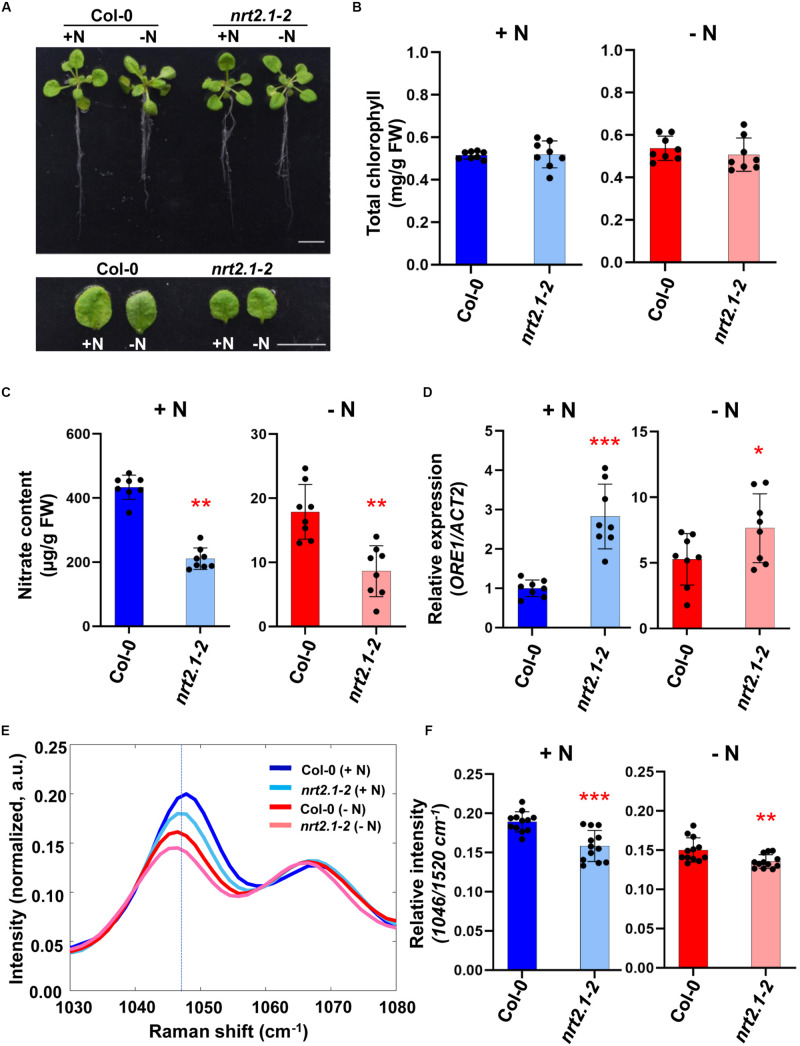
Comparative analysis of biological/molecular phenotype and Raman spectra of early nitrogen deficiency in Arabidopsis WT *and nrt2.1-2.* Three-week-old seedlings of Arabidopsis WT (Col-0) and *nrt2.1-2* were transferred into +N or –N hydroponic medium and grown for 3 days. **(A)** Morphological phenotype, scale bar, 1 cm. *n* = 8 (biologically independent experiments). **(B)** Total chlorophyll content of leaf No. 4 samples was analyzed in +N and –N plants. Data are mean values, *n* = 8 (biologically independent experiments) and individual data points are shown as overlays. FW; fresh weight. *P* values are shown in Supplementary Table S4. **(C)** Nitrate content of leaf No. 4 samples was analyzed in +N and –N plants. Data are mean values, *n* = 8 (biologically independent experiments) and individual data points are shown as overlays. Asterisks indicate statistically significant difference compared with + N. *P < 0.05, **P < 0.01, and ***P < 0.001; two-tailed *t*-test. FW; fresh weight. Supplementary Table S4 shows *P* values. **(D)** *ORE1* transcript levels were analyzed by qRT-PCR in leaf No.4 samples of +N and –N plants Data are mean values, *n* = 8 (biologically independent experiments) and individual data points are shown as overlays. Asterisks indicate statistically significant difference compared with + N. *P < 0.05, **P < 0.01, and ***P < 0.001; two-tailed *t*-test. Supplementary Tables S1, S4 show primer sets and *P* values, respectively. **(E,F)** Leaf No.4 samples of +N or –N plants were measured by Raman spectroscopy. The 1046 cm^–1^ region of Raman spectrum shows the nitrate peak of WT or *nrt2.1-2* in +N or –N condition. Data are mean values, *n* = 12 (biologically independent experiments) and individual data points are shown as overlays. Asterisks indicate statistically significant difference compared with Col-0 (+N). *P < 0.05, **P < 0.01, and ***P < 0.001; two-tailed *t*-test. *P* values were shown in Supplementary Table S5. a.u; arbitrary unit.

In Arabidopsis, several *NRT2* genes are significantly expressed in roots and up-regulated by nitrogen deficiency suggesting that they may be responsible for the stimulation of the nitrate high affinity transporter system under nitrogen limiting conditions (Gansel et al., 2001; Orsel et al., 2002; Okamoto et al., 2003; Ju et al., 2009). Previously, it was shown that the influx capacity of the *nrt2.1*/*nrt2.2* double mutants [named by *nrt2.1-1* in *Wassilewskija* (Ws) and *nrt2.1-2* in Col-0] at low nitrate concentration was decreased. The nitrate influx in *nrt2.1*/*nrt2.2* was consistently reduced more than that in *nrt2.1* at low and high nitrate concentrations (Li et al., 2007). Using *nrt2.1-2* mutant, we analyzed the response of nitrate deficiency. First, we confirmed the genotype of the double mutant *nrt2.1-2* by checking the expression of *NRT2.1* and *NRT2.2* using qRT-PCR (Supplementary Figure S2). We then analyzed molecular phenotypes and Raman spectra in 3-week-old wild-type (Col-0) and *nrt2.1-2* mutant plants grown for 3 days under nitrogen deficient- and sufficient conditions. There was no difference in the phenotypes of WT and *nrt2.1-2* plants grown under +N or −N condition (Figure 3A) and no significant difference in leaf chlorophyll content was detected (Figure 3B). However, chemical analysis showed that the nitrate content of *nrt2.1-2* plants was 2-fold less compared to WT under both +N or −N condition (Figure 3C). Moreover, under +N condition *ORE1* transcript levels in *nrt2.1-2* were slightly induced compared to +N WT plants, and under −N condition, its transcript level of *nrt2.1-2* plants were 5-fold higher and 3-fold higher than WT and *nrt2.1-2* under +N condition, respectively (Figure 3D). These results show that *nrt2.1-2* plants were already under moderate nitrogen deficient stress in +N condition. Figures 3E,F show that the relative peak intensity at 1046 cm^–1^ in the *nrt2.1-2* mutant was significantly lower than in WT under both +N and −N conditions. These changes in 1046 cm^–1^ intensity correlate with changes in nitrate content. Wider-range spectra of Figure 3E are presented in Supplementary Figure S3.

**FIGURE 4:**
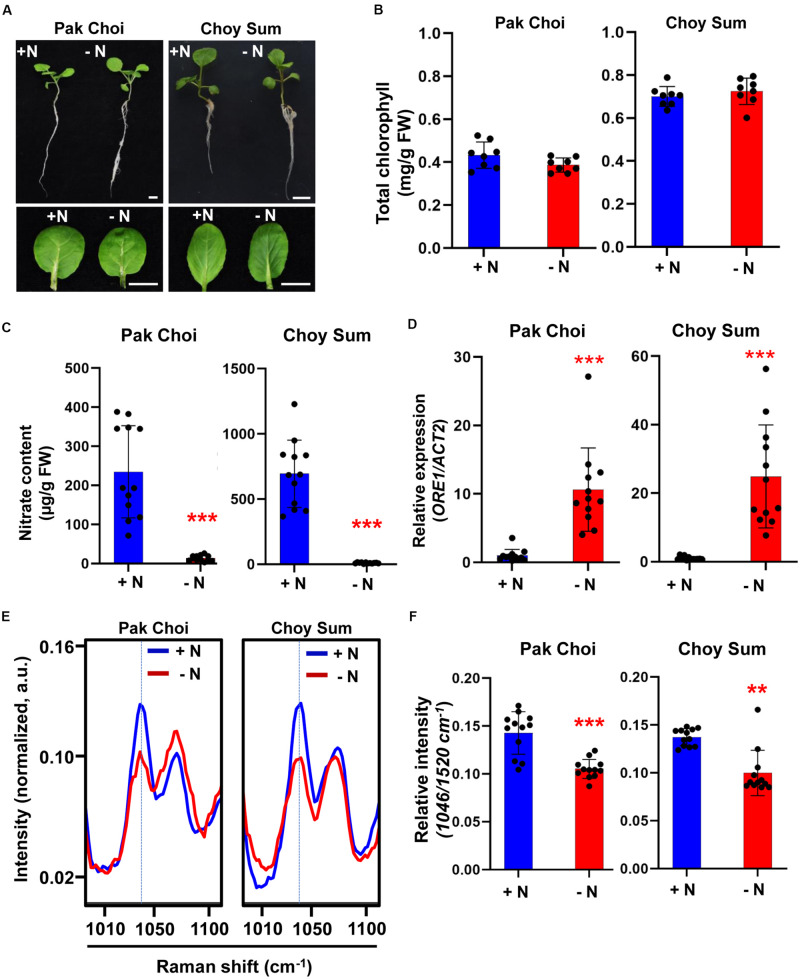
Biological/molecular phenotype and Raman spectral analysis of early nitrogen deficiency in leafy vegetables, Pak Choi and Choy Sum. Two-week-old seedlings of Pak Choi (*Brassica rapa chinensis*) and Choy Sum (*Brassica rapa var. parachinensis*) were transferred into +N or –N hydroponic medium and grown for 5 days. **(A)** Morphological phenotype, scale bar, 1 cm. *n* = 8 (biologically independent experiments). **(B)** Total chlorophyll content of leaf No. 4 samples was analyzed in +N and –N plants. Data are mean values, *n* = 8 (biologically independent experiments) and individual data points are shown as overlays. Asterisks indicate statistically significant difference compared with + N. *P < 0.05, **P < 0.01, and ***P < 0.001; two-tailed *t*-test. FW; fresh weight. *P* values are shown in Supplementary Table S2. **(C)** Nitrate content of leaf No. 4 samples was analyzed in +N and –N plants. Data are mean values, *n* = 12 (biologically independent experiments) and individual data points are shown as overlays. Asterisks indicate statistically significant difference compared with + N. *P < 0.05, **P < 0.01, and ***P < 0.001; two-tailed *t*-test. FW; fresh weight. *P* values are shown in Supplementary Table S2. **(D)** *ORE1* orthologous gene transcript levels were analyzed by qRT-PCR in leaf No. 4 samples of Pak Choi and Choy Sum grown under +N or –N condition for 5 days. Data are mean values, *n* = 12 (biologically independent experiments) and individual data points are shown as overlays. Asterisks indicate statistically significant difference compared with +N. *P < 0.05, **P < 0.01, and ***P < 0.001; two-tailed *t*-test. Supplementary Tables S1, S2 show primer sets and *P* values, respectively. **(E,F)** Leaf No. 4 samples from +N and -N plants were measured by Raman spectroscopy. Only the 1046 cm^–1^ of Raman shift (cm^–1^) is shown. Data are mean values, *n* = 12 (biologically independent experiments) and individual data points are shown as overlays. Asterisks indicate statistically significant difference compared with +N. *P < 0.05, **P < 0.01, and ***P < 0.001; two-tailed *t*-test. *P* values were shown in Supplementary Table S5. a.u; arbitrary unit.

To see if the Raman nitrate peak identified using Arabidopsis can be extended to crop plants, we analyzed Raman spectra of two leafy vegetables belonging to the *Brassicacea* family: Pak Choi (*Brassica rapa chinensis*) and Choy Sum (*Brassica rapa var. parachinensis*). As in Arabidopsis, two varieties of leafy vegetable plants were grown under +N or −N conditions but for 5 days. Similar to Arabidopsis, the two leafy vegetable plants exhibited little phenotypic differences when grown under +N or −N (Figure 4A); neither was there a significant change in their leaf chlorophyll content (Figure 4B). However, nitrate content was significantly decreased in −N plants (Figure 4C). Under −N condition, *ORE1* orthologous gene transcript levels in Pak Choi and Choy Sum were increased by 10- and 20-fold, respectively indicating the implementation of nitrogen stress responses (Figure 4D). In both leafy vegetables, the relative intensity of the 1046 cm^–1^ peak under −N condition was significantly lower than that of +N condition and the peak pattern was similar to that of WT Arabidopsis under −N condition (Figures 4E,F). Wider-range spectra of Figure 4E are shown in Supplementary Figure S4. Taken together, our results suggest that the 1046 cm^–1^ nitrate peak identified by Raman spectroscopy can also be used to diagnose nitrogen status in crop plants as well.

**FIGURE 5:**
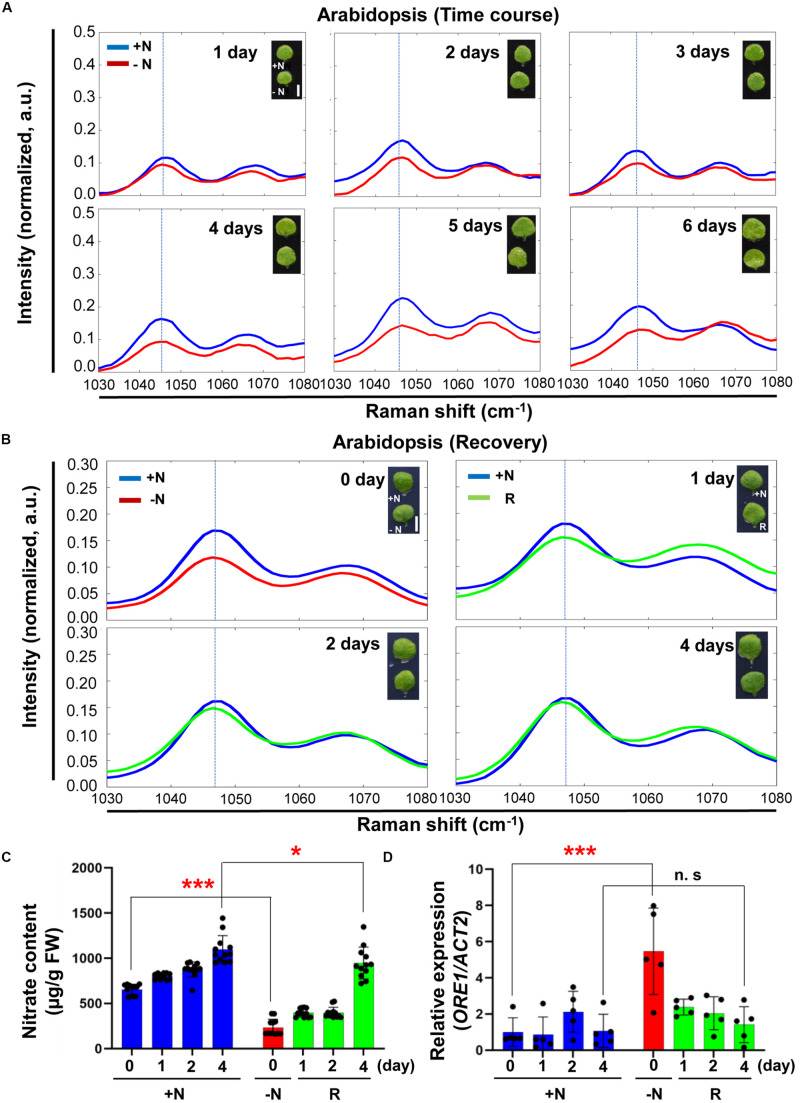
Time course analysis of Arabidopsis under + N, –N and recovery conditions by Raman spectroscopy. **(A)** Three-week-old seedlings of WT (Col-0) were transferred into +N or –N hydroponic medium. Leaf No. 4 samples were measured by Raman spectroscopy at various time points after transfer to –N medium. *n* = 10 (biologically independent experiments). **(B)** Arabidopsis plants grown under –N for 3 days were transferred into +N medium. Plants samples (R) at 0 day were same with samples grown for 3 days under –N medium. Samples were taken at various time points for 4 days. R; plants in the recovery +N medium, Scale bar, 0.5 cm **(A,B)**. a.u; arbitrary unit. *n* = 10 (biologically independent experiments). **(C)** Nitrate content of leaf No. 4 was analyzed in +N or recovery plants (R). Data are mean values, *n* = 12 (biologically independent experiments) and individual data points are shown as overlays. Asterisks indicate statistically significant difference compared with +N. *P < 0.05, **P < 0.01, and ***P < 0.001; two-tailed *t*-test. FW; fresh weight. Supplementary Table S2 shows *P* values. **(D)** *ORE1* transcript levels were analyzed by qRT-PCR in samples (leaf #4) of +N or recovery plants (R). Data are mean values, *n* = 5 (biologically independent experiments) and individual data points are shown as overlays. Asterisks indicate statistically significant difference compared with +N. *P < 0.05, **P < 0.01, and ***P < 0.001; two-tailed *t*-test. Supplementary Tables S1, S6 show primer set and *P* values, respectively.

To see if Raman spectroscopy can be integrated into the management of plant nutritional status, we performed time course experiments of Arabidopsis under −N conditions. Figure 5A shows that the 1046 cm^–1^ peak intensity decreased with time upon transfer to the −N medium. To see if this decrease in peak intensity at 1046 cm^–1^ can be reversed we transferred −N (day 3) plants to +N medium and followed their recovery for several days. Figure 5B shows after one day in the +N medium nitrate peak intensity at 1046 cm^–1^ was still lower than that under nitrogen sufficient condition, but the peak intensity returned to the level of that of +N plants after 4 days in the full medium. Supplementary Figures S5, S6 show the corresponding wider-range spectra of Figures 5A,B, respectively.

We measured nitrate content and analyzed *ORE1* transcript level of plants undergoing recovery in the full medium (Figures 5C,D). Plants starved for nitrate in the −N medium for 3 days were used for the recovery experiment. Compared with +N plants, the nitrate content of these −N plants was decreased by 3-fold. However, the nitrate content returned to the +N levels after 4 days in the +N medium. Changes in the nitrate content were matched by corresponding changes in the nitrate peak intensity (Figure 5C). Because of the nitrate replenishment, *ORE1* transcript levels decreased by 3-fold after 4 days in the recovery medium (Figure 5D).

**FIGURE 6:**
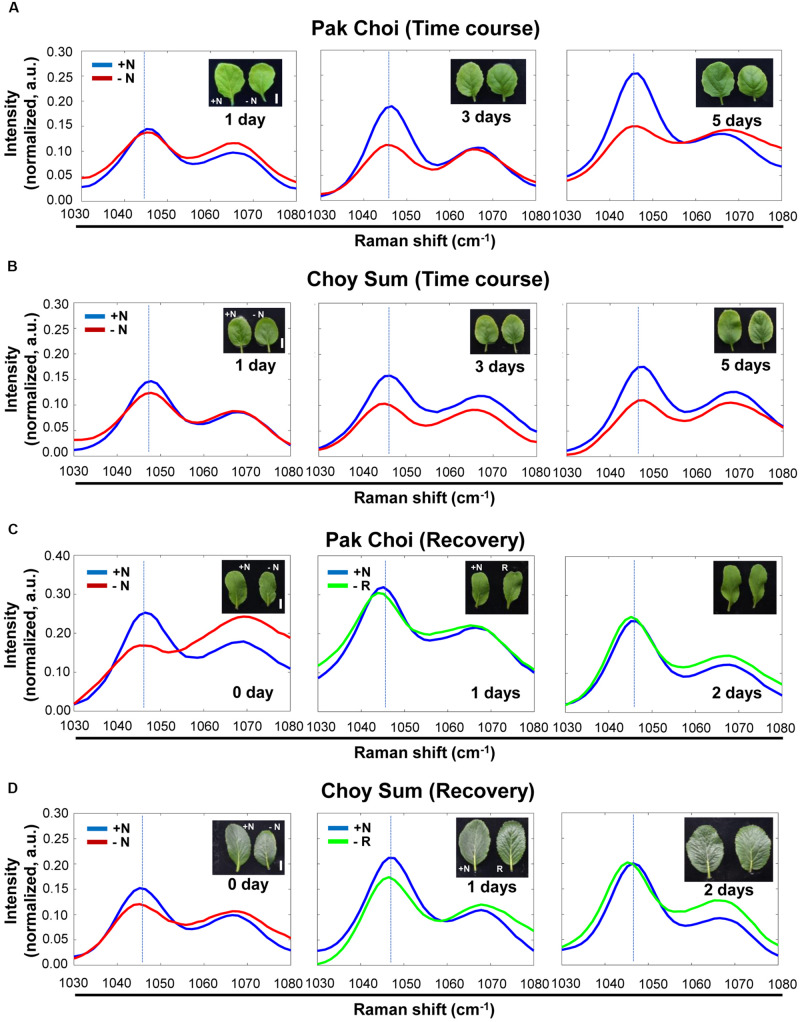
Time course analysis of leafy vegetables, Pak Choi and Choy Sum under + N, –N and recovery conditions by Raman spectroscopy. **(A,B)** Three-week old seedlings of two-week old seedlings of Pak Choi (*Brassica rapa chinensis*) and Choy Sum (*Brassica rapa var. parachinensis*) were transferred into +N or –N hydroponic medium. Leaf No. 4 samples were measured by Raman spectroscopy at various time points after transfer to –N medium. *n* = 10 (biologically independent experiments). **(C,D)** Pak Choi and Choy Sum plants grown under –N for 3 days were transferred into +N medium. Plants samples (R) at 0 day were same with samples grown for 3 days under –N medium. Samples were taken at various time points for 3 days. *n* = 5 (biologically independent experiments). R; recovery plant, Scale bar, 0.5 cm. a.u; arbitrary unit.

Similar experiments with the two leafy vegetable plants (Pak Choi and Choy Sum) showed that the intensity of the 1046 cm^–1^ peak also decreased after 3 and 5 days in the −N medium compared with 1 day in the same medium (Figures 6A,B).

To see if this decrease in peak intensity at 1046 cm^–1^ can be reversed we transferred −N (day 3) plants to +N medium and followed their recovery for several days. Figures 6C,D show after 1 day in the +N recovery medium the nitrate peak intensity at 1046 cm^–1^ was still lower than that of plants under continuous +N condition. However, the peak intensity returned to the level of that of +N plants after 3 days of recovery in the +N medium. Supplementary Figures S7–S10 shows the corresponding wider-range spectra of Figures 6A–D, respectively.

**FIGURE 7:**
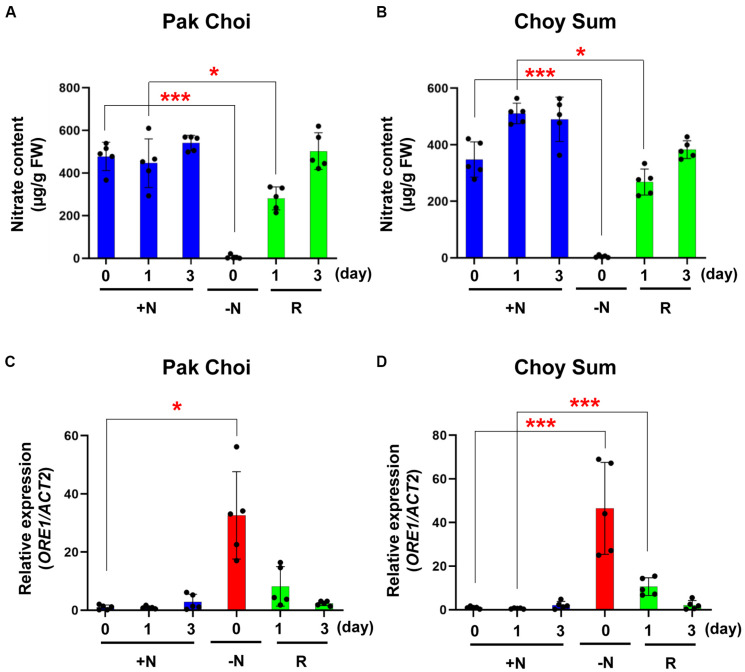
Nitrate content and *ORE1* orthologous gene expression levels of two leafy vegetables in recovery experiments. **(A,B)** Nitrate content of leaf No. 4 was analyzed in +N or recovery plants (R) in Pak Choi **(A)** and Choy Sum **(B)**. Data are mean values, *n* = 5 (biologically independent experiments) and individual data points are shown as overlays. Asterisks indicate statistically significant difference compared with +N. *P < 0.05, **P < 0.01, and ***P < 0.001; two-tailed *t*-test. FW; fresh weight. **(C,D)** *ORE1* orthologous gene transcript levels were analyzed by qRT-PCR in leaf No. 4 samples of +N or recovery plants (R) in Pak Choi **(C)** and Choy Sum **(D)**. Data are mean values, *n* = 5 (biologically independent experiments) and individual data points are shown as overlays. Asterisks indicate statistically significant difference compared with +N. *P < 0.05, **P < 0.01, and ***P < 0.001; two-tailed *t*-test. Supplementary Tables S1, S7 show primer sets and *P* values, respectively.

We measured nitrate content and analyzed *ORE1* orthologous gene transcript levels of the two leafy vegetable plants undergoing recovery in the full medium (Figure 7). Compared with plants under continuous +N condition, the nitrate content of these −N Pak Choi and Choy Sum plants was decreased by 500- and 350-fold, respectively, but it returned to the +N level after 3 days in the +N medium. Changes in the nitrate content of the two leafy vegetables were paralleled by corresponding changes in the nitrate peak intensity (Figures 7A,B). Similar results were obtained with Arabidopsis *ORE1* transcript levels (Figures 7C,D).

We performed Principal Component Analysis on the Raman spectra of +N and −N plants and separation between groups was observed for both Arabidopsis and Pak Choy (Supplementary Figures S11A, S12A). The strongest Raman spectral lines in the data set, which are attributed to carotenoids, were also observed in PC1 and were responsible for the largest spectral variance. The variance corresponding to PC2 can be used to partition the Raman spectra into classes relating to +N and -N conditions. The presence of the peak at 1046 cm^–1^ – which we identified as a nitrate Raman peak - in PC2 demonstrated that this region of the Raman spectra represented one of the main differentiation factors for +N and −N plants (Supplementary Figures S11B, S12B).

## Discussion

Raman spectroscopy has been used to analyse either chlorophyll *a* or β-carotene content in microalgae under nitrogen deficient conditions (Heraud et al., 2006) and changes in storage lipids were clearly identified by Raman spectroscopy in nitrogen deficient *C. sorokiniana* and *N. oleoabundans* (Huang et al., 2010). Several well-identified Raman peaks corresponding to chlorophylls, carotenoids and triglycerides have been reported (Baranski et al., 2005; Pudney et al., 2011; Altangerel et al., 2017). However, changes in the intensity of these peaks are also seen in plants experiencing biotic and abiotic stresses rendering them unsuitable for use as specific Raman signatures for plants experiencing nitrogen deficiency. Near-infrared hyperspectral imaging has been used to diagnose nitrogen deficiency in cucumber plants based on chlorophyll distribution map of the plant (Shi et al., 2012). The drawback of this method is that it can only be used at the late stage of nitrogen deficiency when chlorophyll degradation occurs; besides, it is not specific to nitrogen stress because chlorophyll degradation can also be induced by many biotic and abiotic stresses.

Using standard solutions containing different concentrations of sodium nitrate Mabrouk et al. (2013) reported nitrate Raman shift of 1020 to 1070 cm^−1^. In addition, Chirico et al. (2016) showed that the Raman peak at 1043 cm^−1^, assigned to the totally symmetric vibration of NO_3_ ions, could be clearly measured in ammonium nitrate. Our results have confirmed these previous observations (Irish and Walrafen, 1967; Mabrouk et al., 2013; Chirico et al., 2016).

Here, we show that Raman spectroscopy can be used to query the state of plant health in a non-invasive manner and we have identified the 1046 cm^–1^ Raman peak as a specific signature of nitrogen status in Arabidopsis and vegetable crops *in planta*.

Five lines of evidence support our claims:

(1) We have confirmed the Raman shift at 1046 cm^−1^ attributed to nitrate using standard chemicals such as calcium nitrate [Ca(NO_3_)_2_], potassium nitrate (KNO_3_) and ammonium nitrate (NH_4_NO_3_).
(2) In WT plants the 1046 cm^–1^ peak intensity correlates with the nitrate content in WT Arabidopsis plants in starvation and recovery experiments.
(3) The peak intensity is reduced in Arabidopsis mutant in *nrt2.1*/*nrt2.2* which is partially blocked in nitrate uptake.
(4) The peak intensity also correlates with nitrate content in two leafy vegetables (Pak Choi and Choy Sum) in starvation and recovery conditions.
(5) This Raman peak is specific to nitrogen stress as its intensity is not altered in plants under -P or -K.

## Conclusion

An important aspect of our work is that nitrate deficiency of Arabidopsis as well as vegetables can be diagnosed by its specific Raman signature as early as 3 days on the starvation medium before the onset of any symptomatic manifestation of the deficient plants. This would facilitate plant stress management through early diagnosis of N deficiency in a non-invasive manner and allow application of appropriate remedial measures to ameliorate the stress. To this end, we have shown that −N plants can recover from nitrogen stress by returning them to a full medium, and along with this recovery the nitrate peak intensity also returns to the +N level. Our results show that Raman spectroscopy can be deployed as a tool for precision agriculture and we anticipate this method will be useful in the field management of crops.

## Data Availability Statement

All datasets generated for this study are included in the article/Supplementary Material.

## Author Contributions

CH, GS, N-HC, RR, and BP designed the experiments. CH, GS, and SP executed the experiments. All of the authors interpreted and discussed the data. CH, GS, N-HC, RR, and BP wrote the manuscript.

## Conflict of Interest

The method for early diagnosis and management of nitrogen deficiency in plants utilizing Raman spectroscopy described in this manuscript has been included in a patent application filed by CH, GS, N-HC, RR, and BP. The remaining author declares that the research was conducted in the absence of any commercial or financial relationships that could be construed as a potential conflict of interest.

## Supplementary Material

FIGURE S1 Schematic of the Raman spectroscopy setup. The excitation laser wavelength of 830 nm and power of 60 mW at the sample was used. The CCD camera was thermoelectrically cooled to −80°C. CCD, charge-coupled devices.

FIGURE S2 Genotyping by qRT-PCR analysis of the *nrt2.1-2* mutant. **(A)** PCR of the knock-out line of *nrt2.1-2* was conducted by left/right genomic primer (LP and RP) and the left T-DNA border primer (LBb1.3). *n* = 3 (biologically independent experiments). **(B)** Expression levels of *NRT2.1* and *NRT2.2* were analyzed by qRT-PCR in WT and mutant plants. Data are mean values ± S.D., *n* = 5 (biologically independent experiments).

FIGURE S3 Comparative Raman spectroscopic analysis of Arabidopsis WT and *nrt2.1-2* under +N or -N conditions. The 900 to 1,600 (cm^–1^) region of the Raman spectrum is shown. For details, see legend to Figure 3E.

FIGURE S4 Raman spectroscopic analysis of leafy vegetables under -N or +N condition. **(A)** Pak Choi; **(B)** Choy Sum. See Figure 4E legend for details.

FIGURE S5 Comparative analysis of wide-range Raman spectrum of Arabidopsis plants grown under +N or -N condition by time course. See Figure 5A legend for details.

FIGURE S6 Comparative analysis of wide-range Raman spectrum of Arabidopsis plants grown for recovery experiments by time course. Plants (R) were first grown in -N condition for 3 days before being returned to +N condition for an additional 3 days. See Figure 5B legend for details.

FIGURE S7 Raman spectroscopic analysis of leafy vegetables, Pak Choi under +N or −N condition by time course. See Figure 6A legend for details.

FIGURE S8 Raman spectroscopic analysis of leafy vegetables, Choy Sum under +N or −N condition by time course. See Figure 6B legend for details.

FIGURE S9 Comparative analysis of wide-range Raman spectrum of leafy vegetables, Pak Choi grown for recovery experiments by time course. See Figure 6C legend for details.

FIGURE S10 Comparative analysis of wide-range Raman spectrum of leafy vegetables, Choy Sum grown for recovery experiments by time course. See Figure 6D legend for details.

FIGURE S11 Principal Component Analysis of Raman spectra acquired from nitrogen sufficient and nitrogen deficient Arabidopsis leaf samples. **(A)** Classification of nitrate sufficient and deficient samples based on the first two principal components (PCs) is shown. **(B)** The principal component 2 (PC2) clearly shows the presence of 1046 cm^–1^ Raman peak which we have identified as nitrate Raman peak in plant leaves.

FIGURE S12 Principal Component Analysis of Raman spectra acquired from nitrogen sufficient and nitrogen deficient Pak Choi leaf samples. **(A)** Classification of nitrate sufficient and deficient samples based on the first two principal components (PCs) is shown. **(B)** The principal component 2 (PC2) clearly shows the presence of 1046 cm^–1^ Raman peak which we have identified as nitrate Raman peak in plant leaves.

TABLE S1 Primer sets for qRT-PCR and genotyping analysis.

TABLE S2 *P*-value data for Figures 1B–D, 4B–D.

TABLE S3 *P*-value data for Figure 2D.

TABLE S4 *P*-value data for Figures 3B–D. ^∗^Nitrate peak indicated Raman spectrum at 1046 cm^–1^ and normalized with cartenoids peak (1520 cm^–1^). *P* value of nutrient-deprived growth (−N, −P, and −K, respectively) relative to Full medium as obtained from student’s *t*-test analysis (*n*=14–16).

TABLE S5 *P*-value data for intensity in Figures 3F, 4F.

TABLE S6 *P*-value data for Figures 5C,D.

TABLE S7 *P*-value data for Figure 7.
