# Supplementary figures and images for: Early Diagnosis and Management of Nitrogen Deficiency in Plants Utilizing Raman Spectroscopy
Source: Front Plant Sci. 2020 Jun 5;11:663. doi: 10.3389/fpls.2020.00663 (PMC7291773; doi:10.3389/fpls.2020.00663)

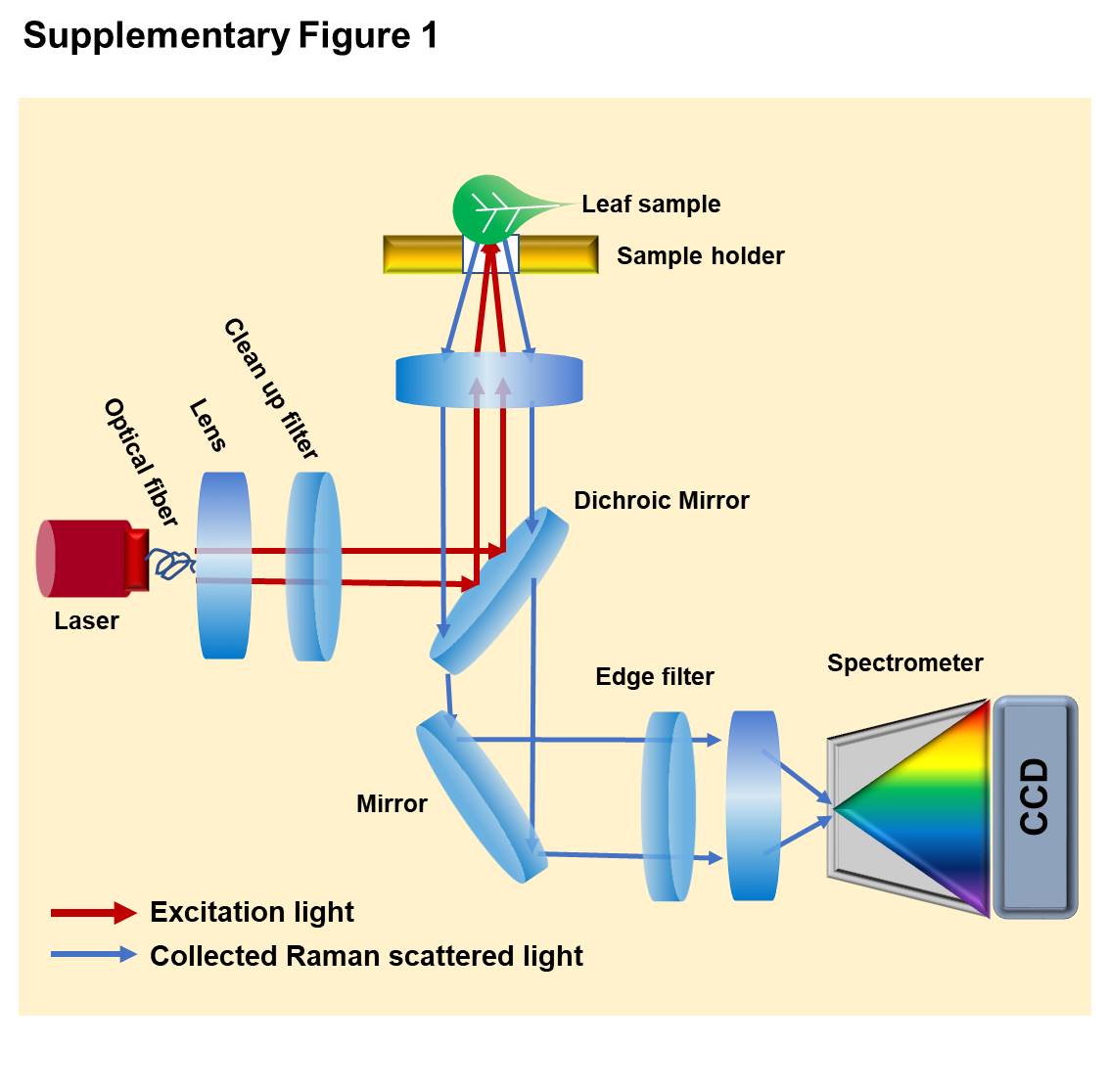

Supplement: FIGURE S1 — Schematic of the Raman spectroscopy setup. The excitation laser wavelength of 830 nm and power of 60 mW at the sample was used. The CCD camera was thermoelectrically cooled to −80°C. CCD, charge-coupled devices. [file Image_1.jpg]

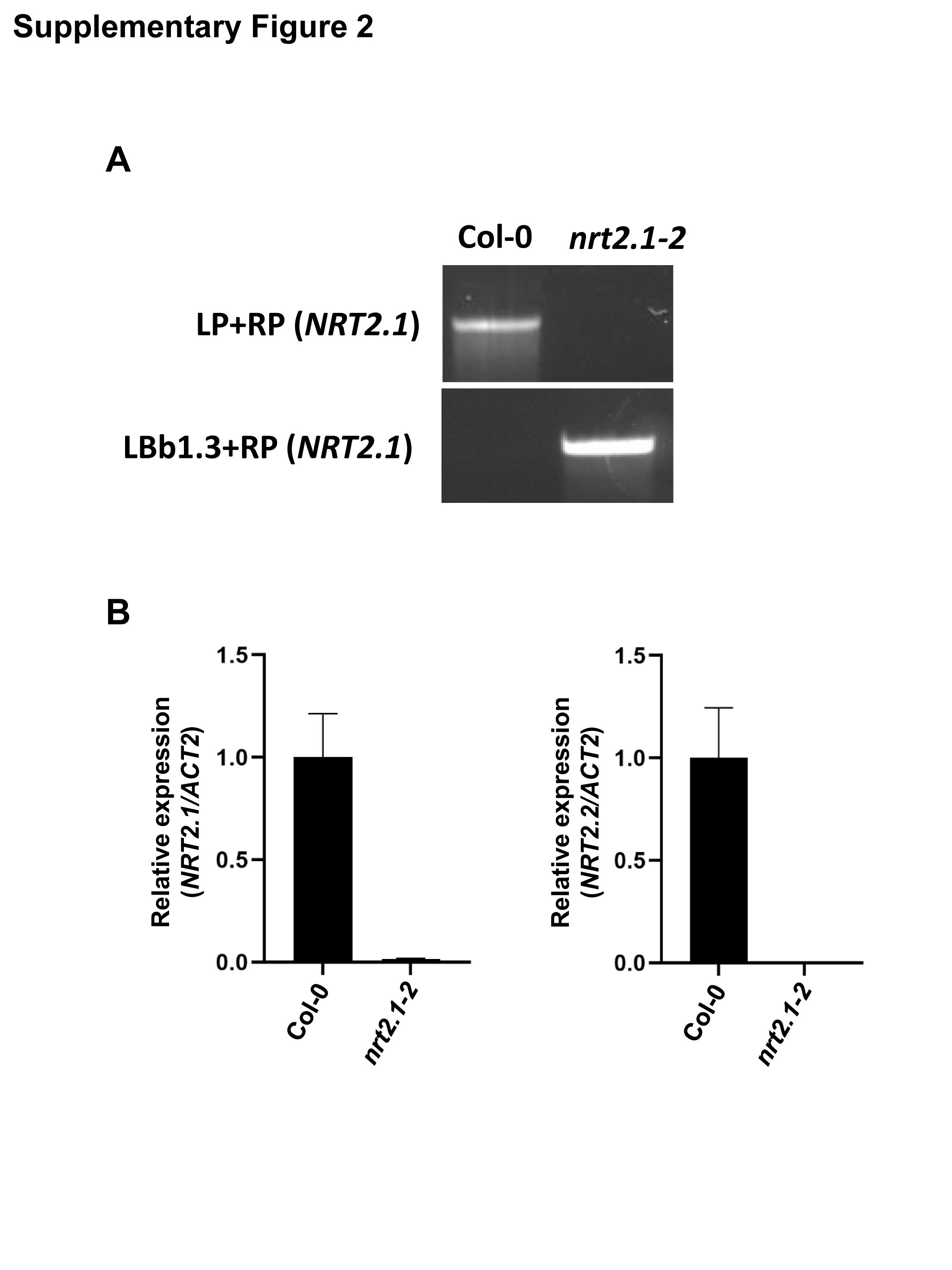

Supplement: FIGURE S2 — Genotyping by qRT-PCR analysis of the nrt2.1-2 mutant. (A) PCR of the knock-out line of nrt2.1-2 was conducted by left/right genomic primer (LP and RP) and the left T-DNA border primer (LBb1.3). n = 3 (biologically independent experiments). (B) Expression levels of NRT2.1 and NRT2.2 were analyzed by qRT-PCR in WT and mutant plants. Data are mean values ± S.D., n = 5 (biologically independent experiments). [file Image_2.jpg]

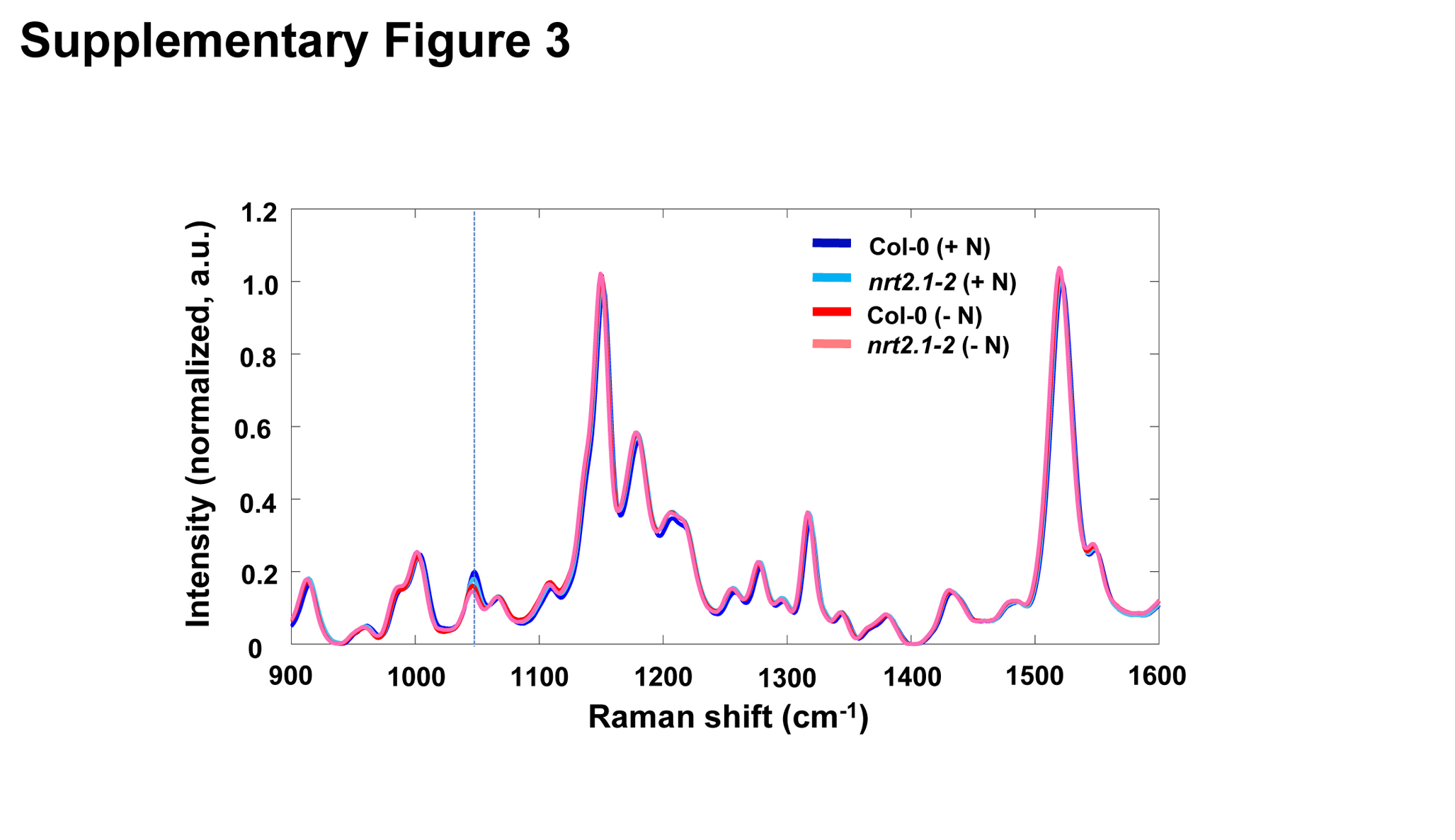

Supplement: FIGURE S3 — Comparative Raman spectroscopic analysis of Arabidopsis WT and nrt2.1-2 under +N or -N conditions. The 900 to 1,600 (cm–1) region of the Raman spectrum is shown. For details, see legend to Figure 3E. [file Image_3.jpg]

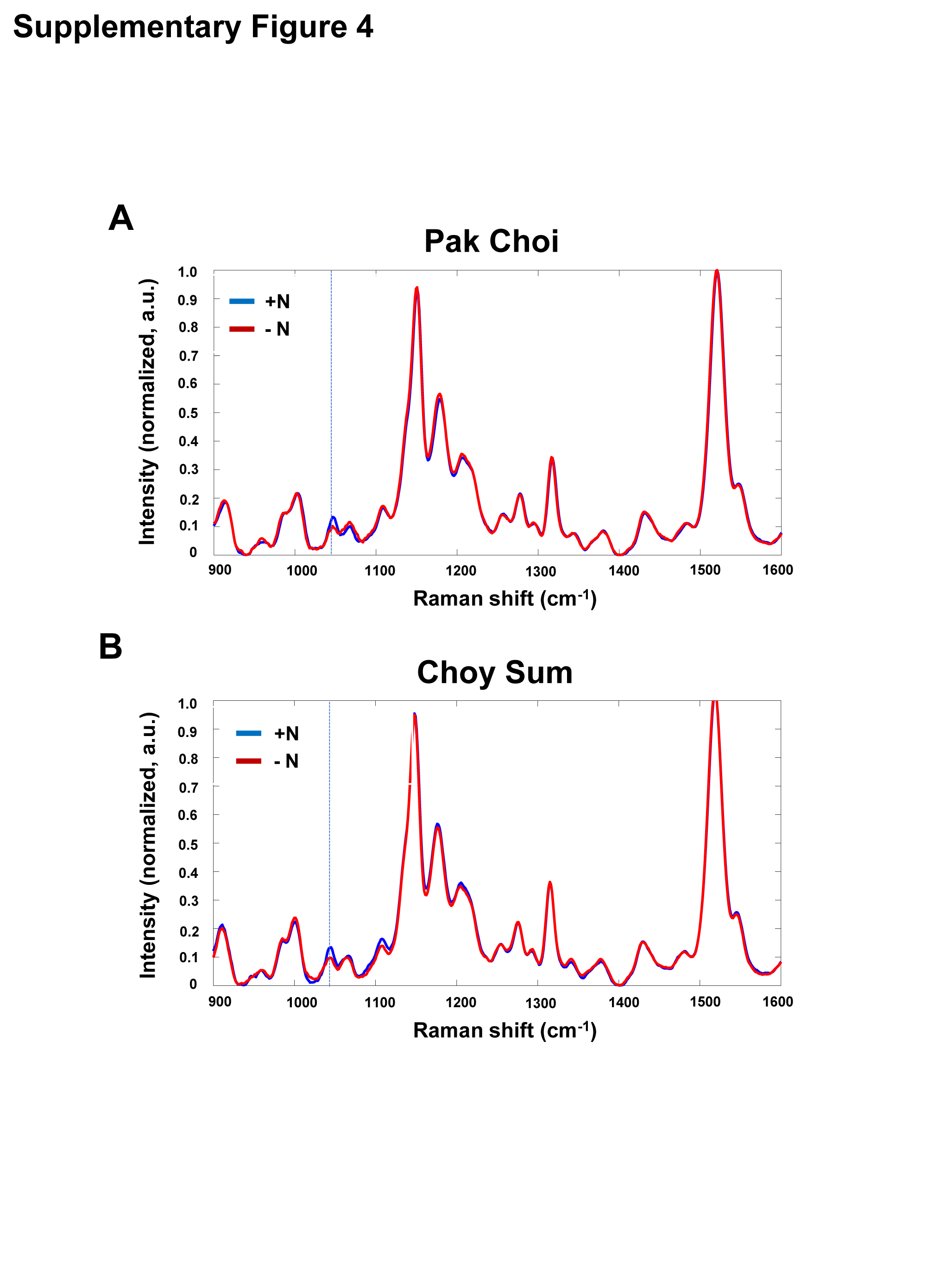

Supplement: FIGURE S4 — Raman spectroscopic analysis of leafy vegetables under -N or +N condition. (A) Pak Choi; (B) Choy Sum. See Figure 4E legend for details. [file Image_4.jpg]

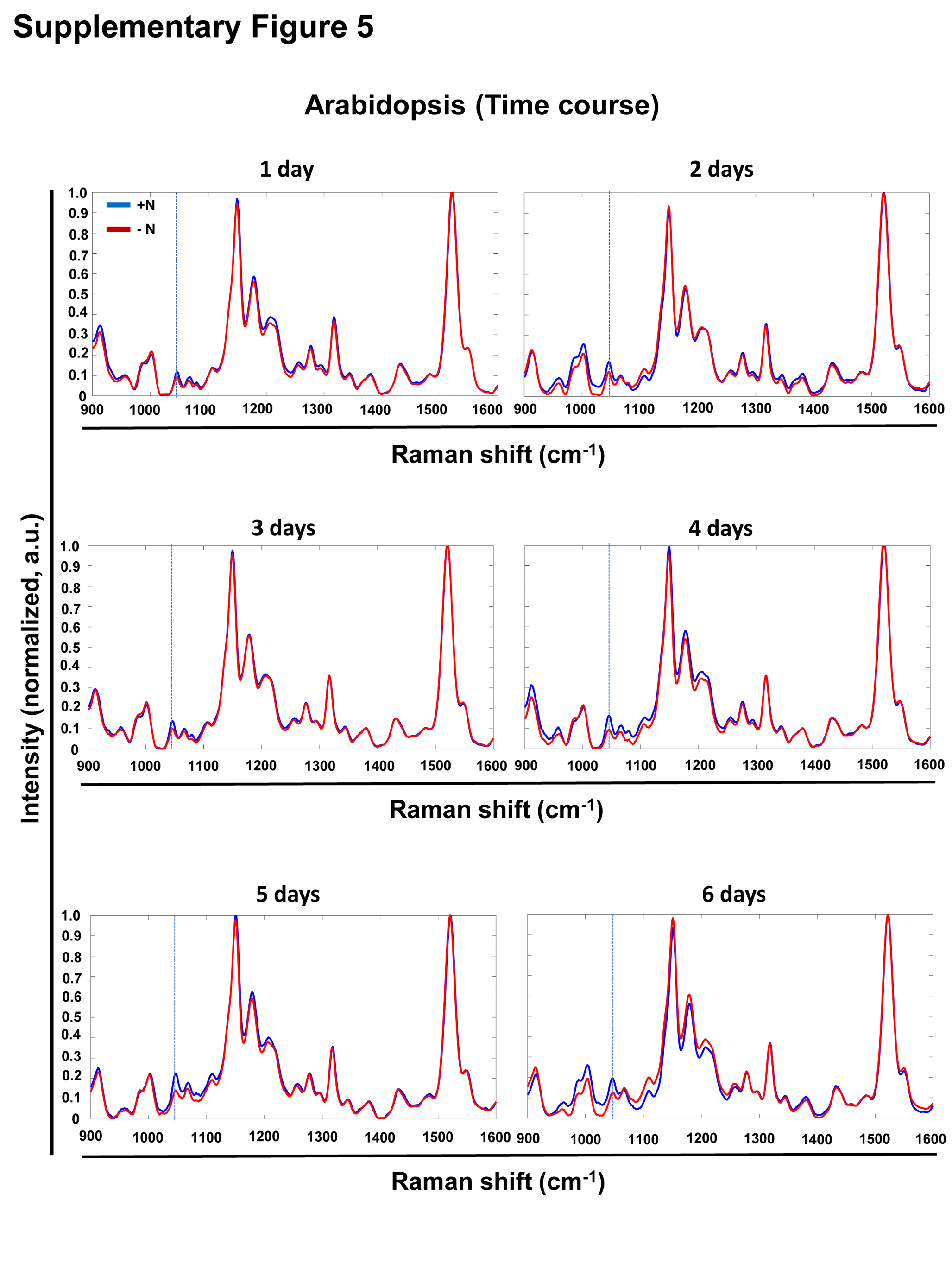

Supplement: FIGURE S5 — Comparative analysis of wide-range Raman spectrum of Arabidopsis plants grown under +N or -N condition by time course. See Figure 5A legend for details. [file Image_5.jpg]

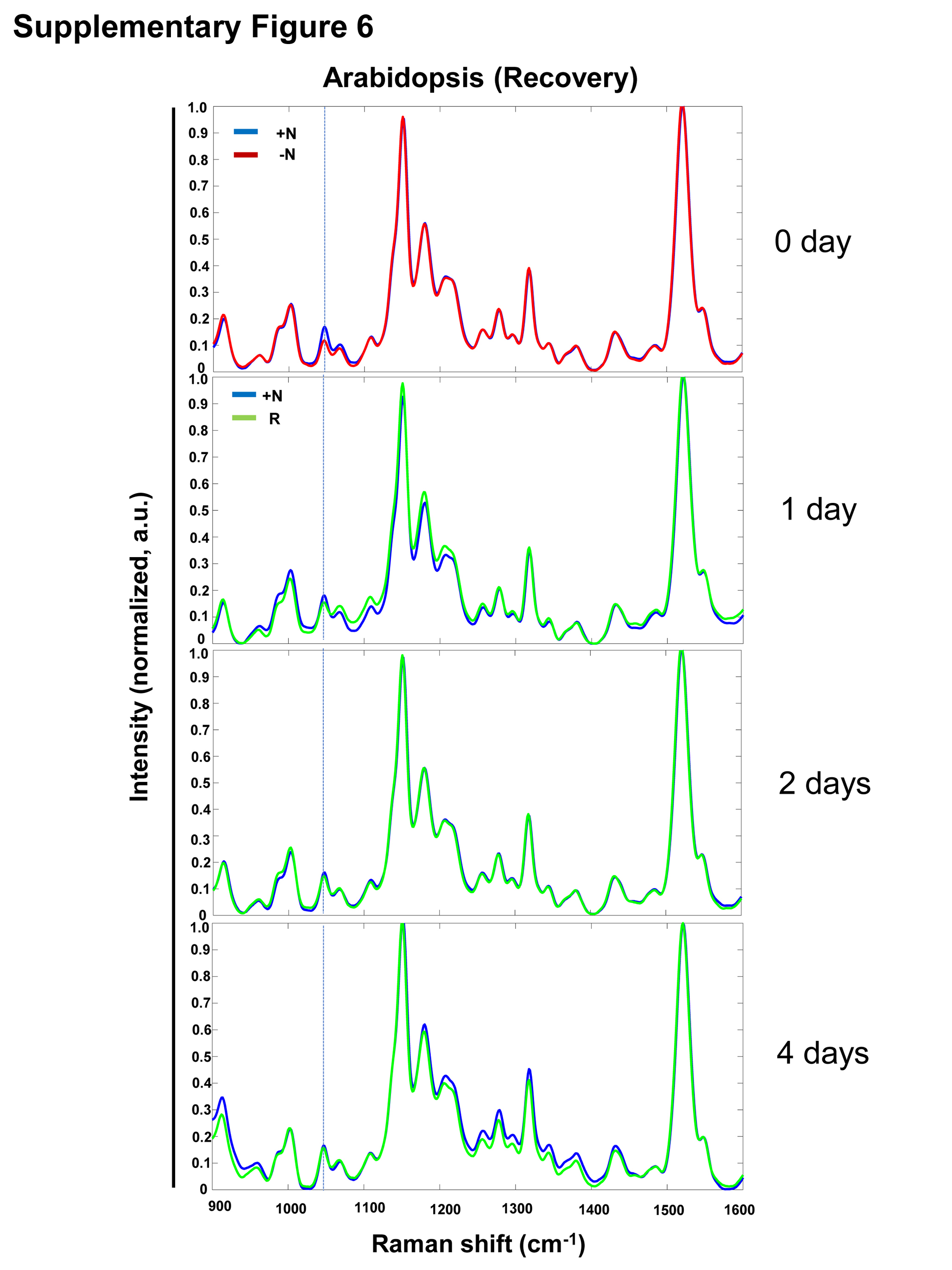

Supplement: FIGURE S6 — Comparative analysis of wide-range Raman spectrum of Arabidopsis plants grown for recovery experiments by time course. Plants (R) were first grown in -N condition for 3 days before being returned to +N condition for an additional 3 days. See Figure 5B legend for details. [file Image_6.jpg]

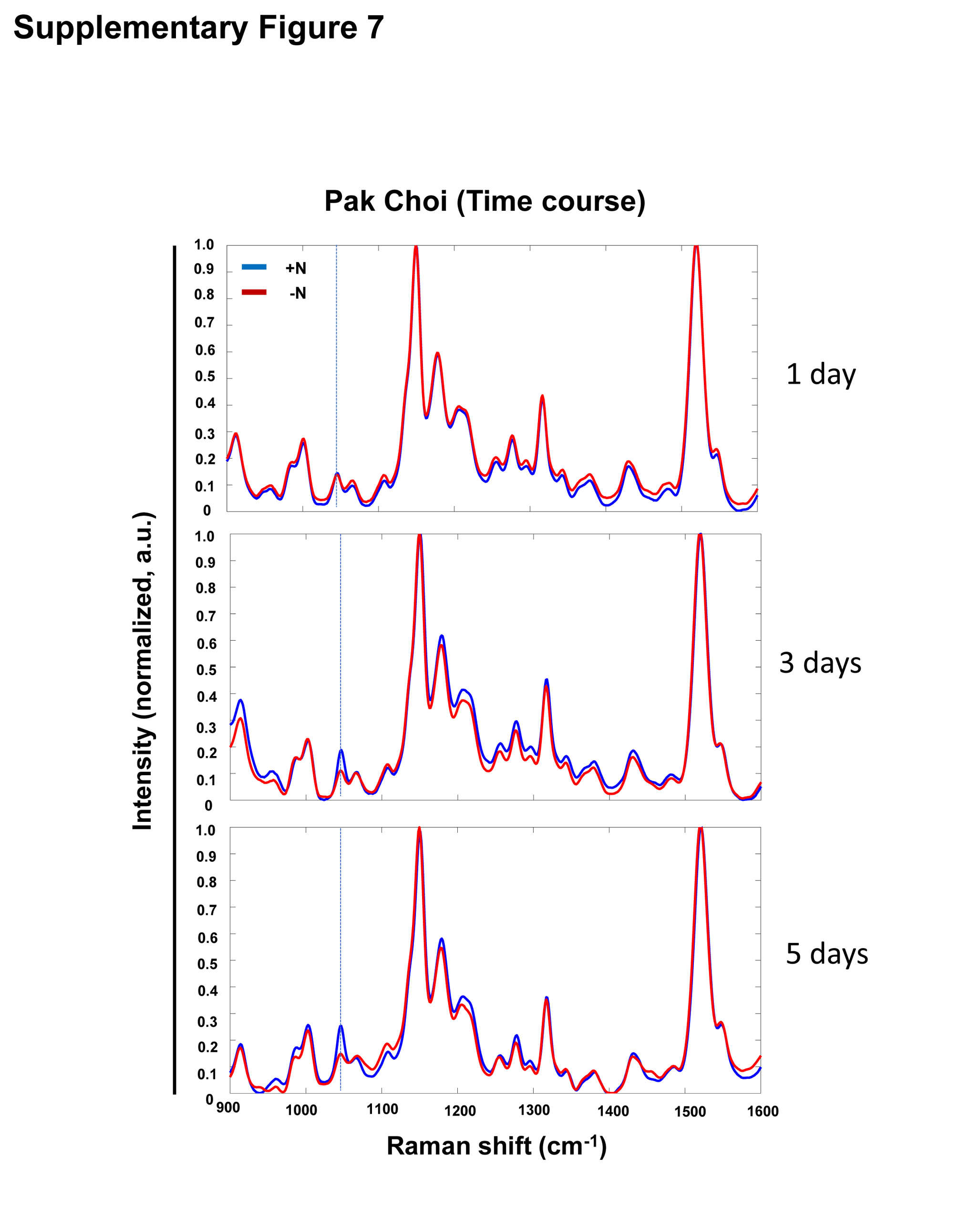

Supplement: FIGURE S7 — Raman spectroscopic analysis of leafy vegetables, Pak Choi under +N or −N condition by time course. See Figure 6A legend for details. [file Image_7.jpg]

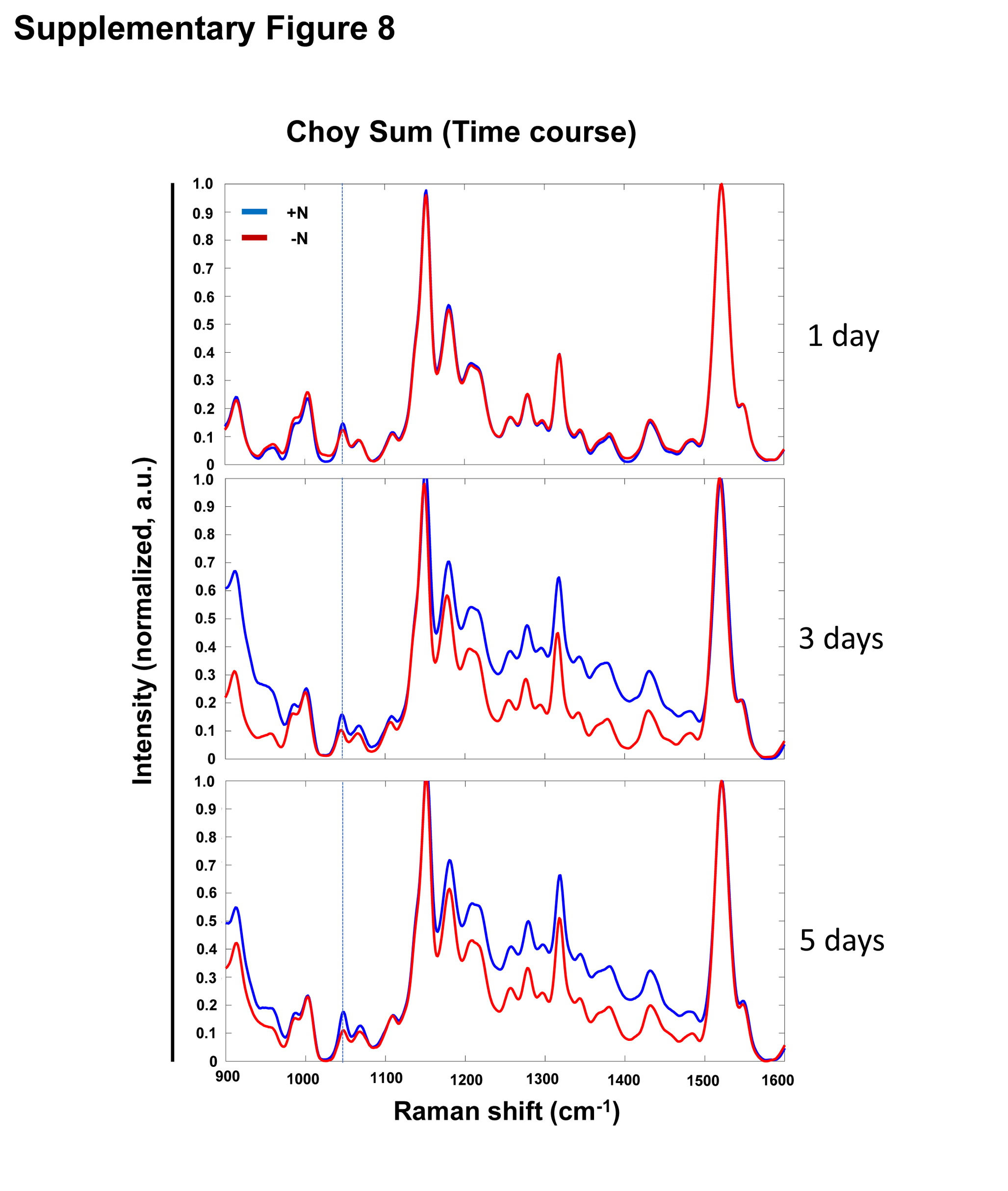

Supplement: FIGURE S8 — Raman spectroscopic analysis of leafy vegetables, Choy Sum under +N or −N condition by time course. See Figure 6B legend for details. [file Image_8.jpg]

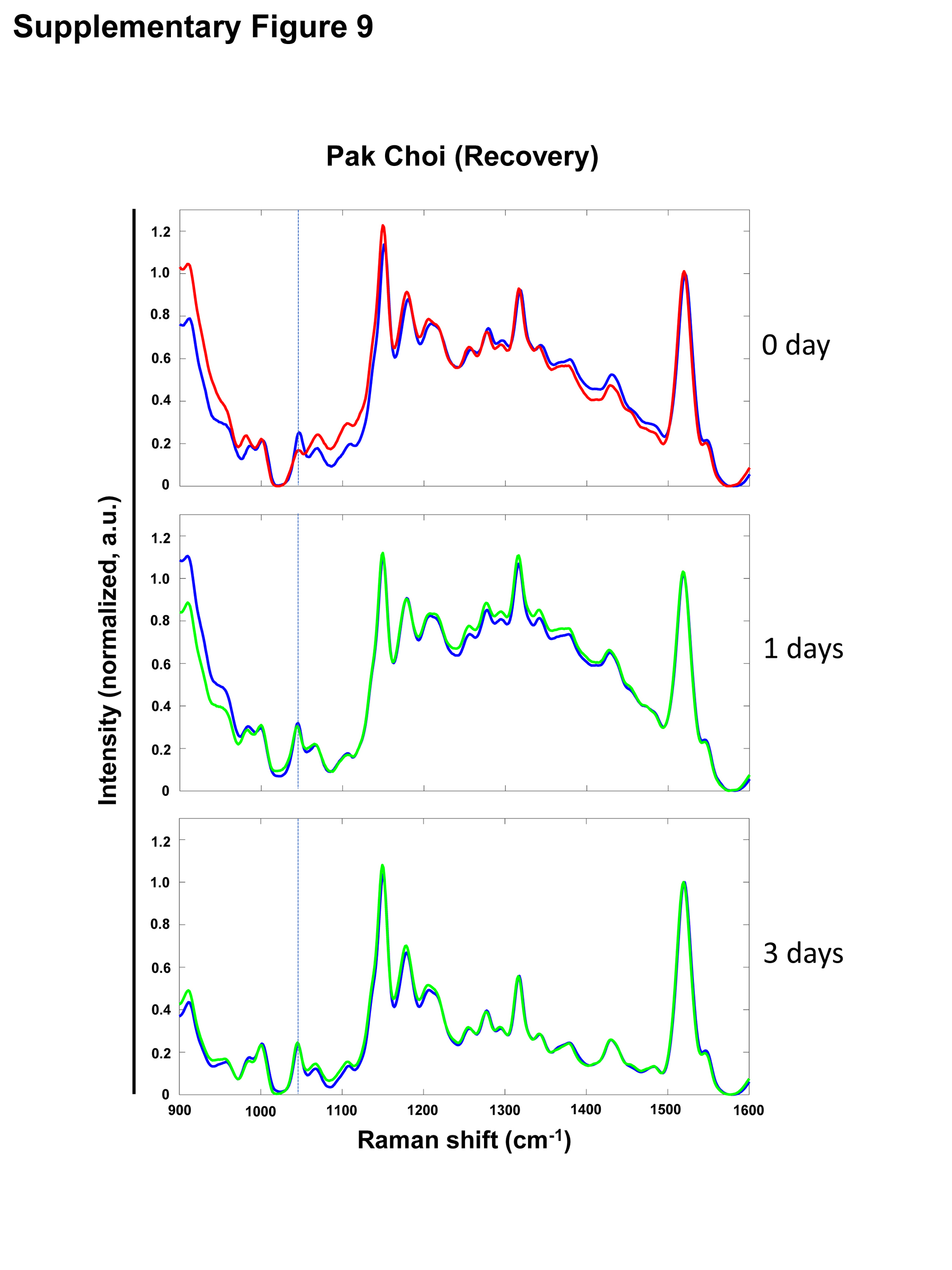

Supplement: FIGURE S9 — Comparative analysis of wide-range Raman spectrum of leafy vegetables, Pak Choi grown for recovery experiments by time course. See Figure 6C legend for details. [file Image_9.jpg]

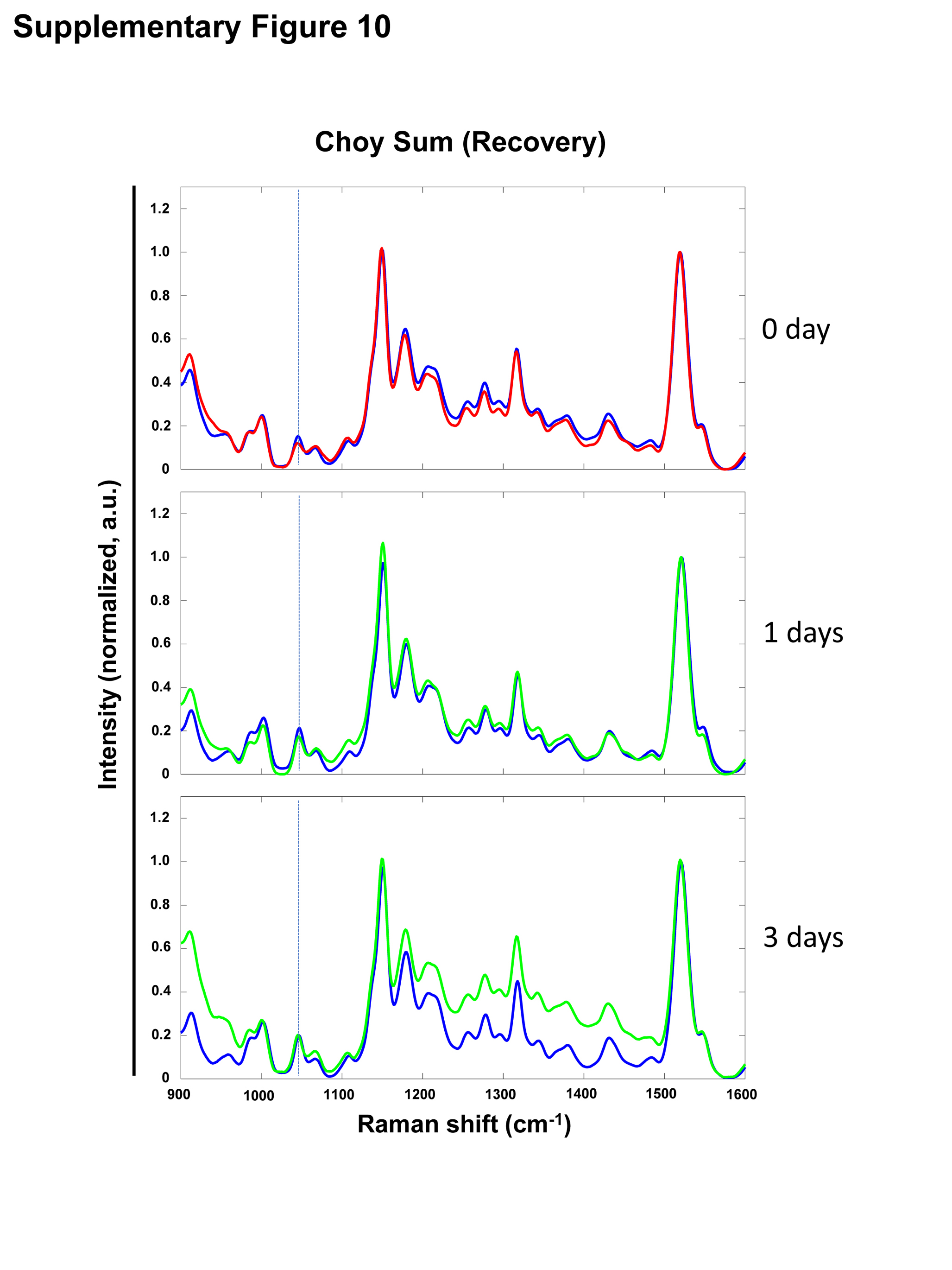

Supplement: FIGURE S10 — Comparative analysis of wide-range Raman spectrum of leafy vegetables, Choy Sum grown for recovery experiments by time course. See Figure 6D legend for details. [file Image_10.jpg]

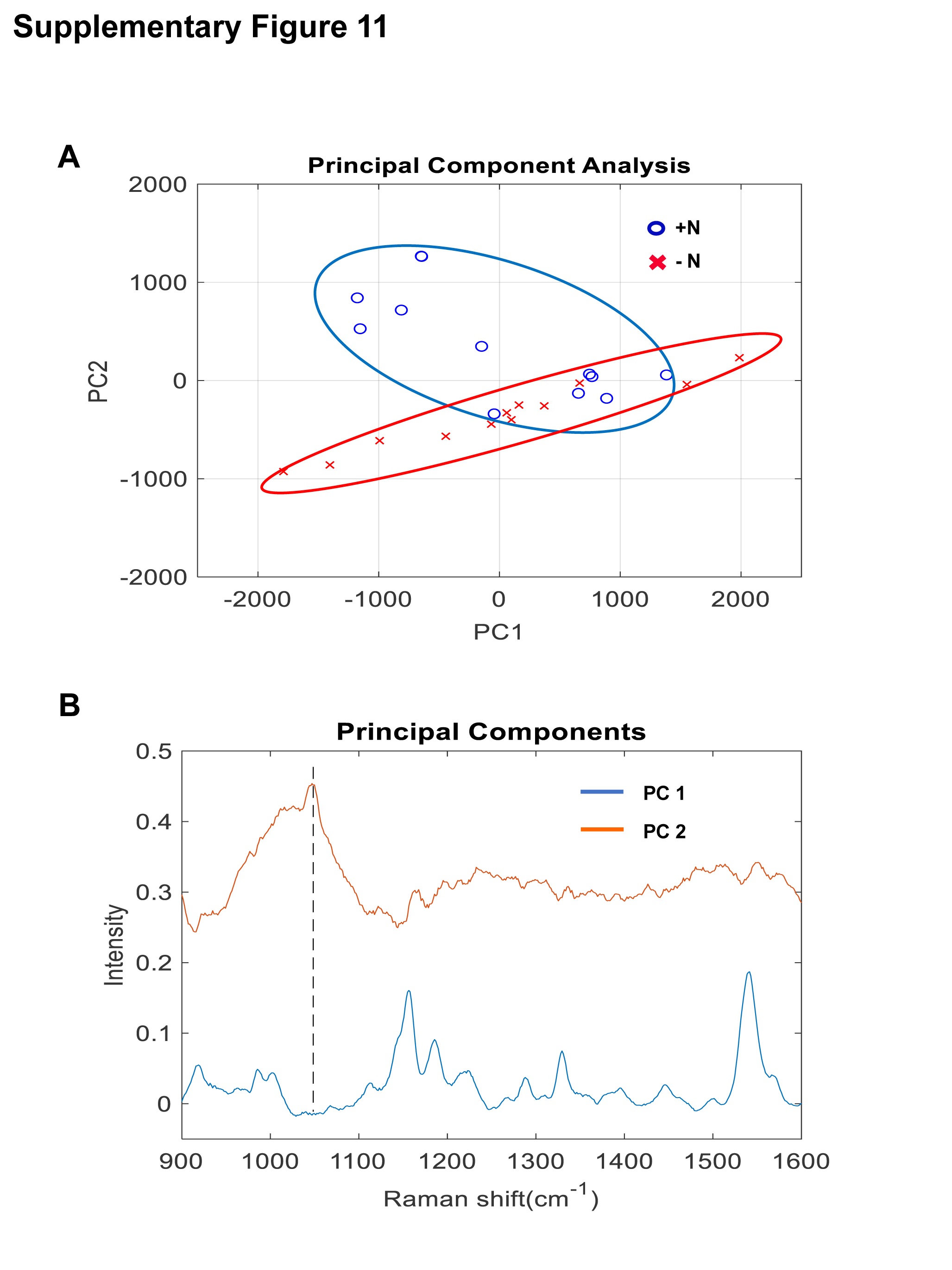

Supplement: FIGURE S11 — Principal Component Analysis of Raman spectra acquired from nitrogen sufficient and nitrogen deficient Arabidopsis leaf samples. (A) Classification of nitrate sufficient and deficient samples based on the first two principal components (PCs) is shown. (B) The principal component 2 (PC2) clearly shows the presence of 1046 cm–1 Raman peak which we have identified as nitrate Raman peak in plant leaves. [file Image_11.jpg]

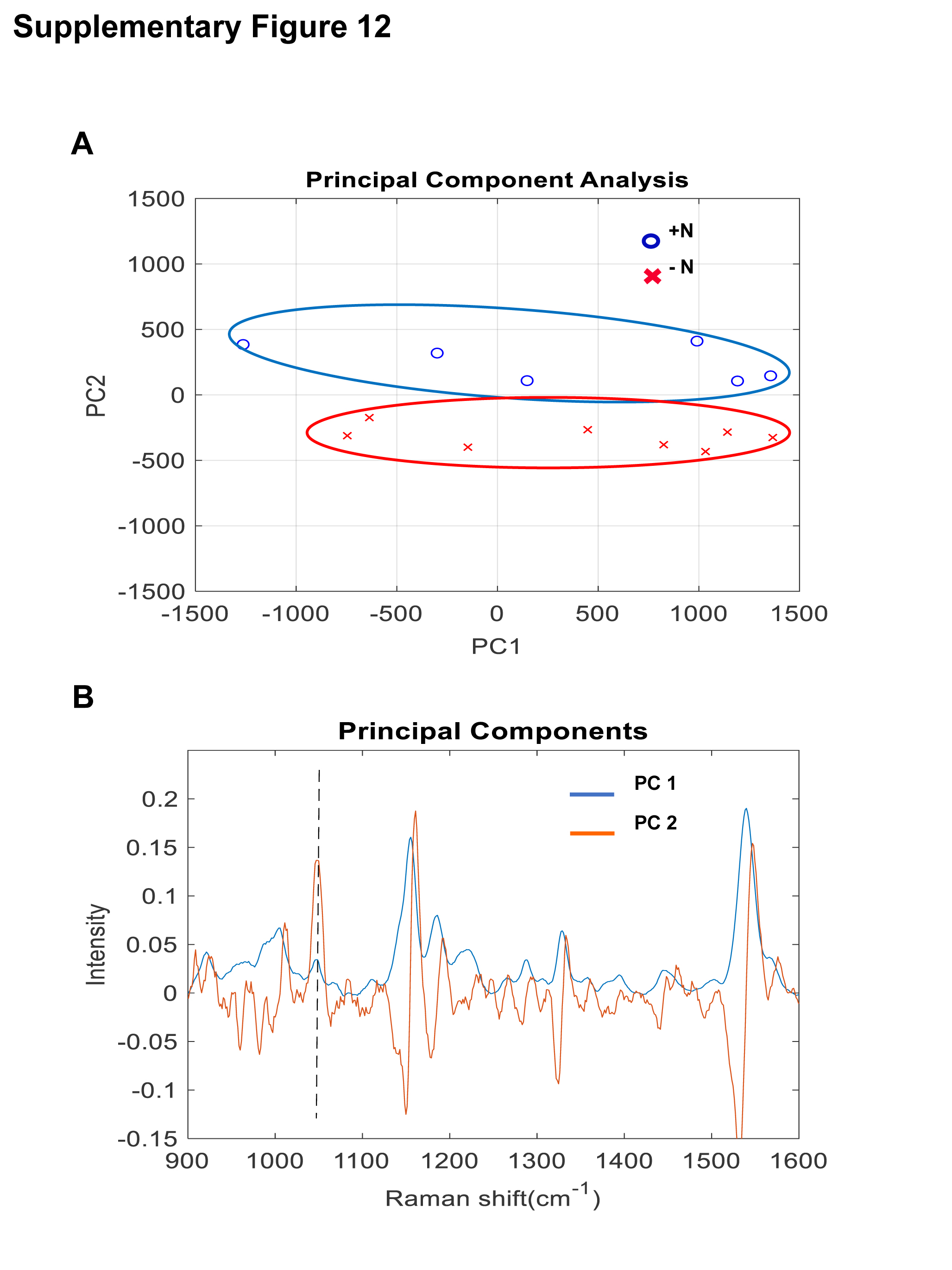

Supplement: FIGURE S12 — Principal Component Analysis of Raman spectra acquired from nitrogen sufficient and nitrogen deficient Pak Choi leaf samples. (A) Classification of nitrate sufficient and deficient samples based on the first two principal components (PCs) is shown. (B) The principal component 2 (PC2) clearly shows the presence of 1046 cm–1 Raman peak which we have identified as nitrate Raman peak in plant leaves. [file Image_12.jpg]
