# Supplementary material for: Early Diagnosis and Management of Nitrogen Deficiency in Plants Utilizing Raman Spectroscopy
Source: Front Plant Sci. 2020 Jun 5;11:663. doi: 10.3389/fpls.2020.00663 (PMC7291773; doi:10.3389/fpls.2020.00663)
Supplement: TABLE S1 — Primer sets for qRT-PCR and genotyping analysis. [file Table_1.pdf]

Supplementary Table1. Primer sets for qRT-PCR and genotyping analysis

Gene name	Primer sequence
**qRT-PCR**	
***ORE1* (Arabidopsis)**	F 5'-CTTACCATGGAAGGCTAAGATGGG -3'<br>R 5'-TCGGGTATTTCCGGTCTCTCAC -3'
***ORE1* (Pak Choi)**	F 5'-CGATGCATCAAGAATCGGTGA -3'<br>R 5'- CGGTGGCAGAGAAGAAAGTG-3'
***ORE1* (Choy Sum)**	F 5'- GGGAAGTCACTTGTGGGTATG -3'<br>R 5'- CTTTGTACCATCGGCACGTT -3'
***NRT2.1*(Arabidopsis)**	F 5'- TGAGCAGGAGAAGCAGAAGA-3'<br>R 5'- TTGTTGGGTGTGTTCTCAGG-3'
***NRT2.2* (Arabidopsis)**	F 5'-GCTATGCTTTCTCGGTAGATGGTAG-3'<br>R 5'-AATGTCATGTTTGGTGAGGTTAAGA-3'
***ACT2* (Arabidopsis)**	F 5'-AGTGGTCGTACAACCGGTATTGT-3'<br>R 5'-GATGGCATGAGGAAGAGAGAAAC -3'
***ACT2* (Pak Choi)**	F 5'- TGCTGGATTCTGGTGATGGT-3'<br>R 5'- GGCGTGTGGAAGAGAGAAAC-3'
***ACT2* (Choy Sum)**	F 5'- TGCTGGATTCTGGTGATGGT-3'<br>R 5'- GGCGTGTGGAAGAGAGAAAC-3'
**Genotyping**	
**LBb1.3**	5'-ATTTTGCCGATTTCGGAAC -3'
**LP (*nrt2.1-2*)**	5'-GTTCTCCATGAGCTTCGTGAG -3'
**RP (*nrt2.1-2*)**	5'-CTTACCATGGAAGGCTAAGATGGG -3'
