# Supplementary material for: Early Diagnosis and Management of Nitrogen Deficiency in Plants Utilizing Raman Spectroscopy
Source: Front Plant Sci. 2020 Jun 5;11:663. doi: 10.3389/fpls.2020.00663 (PMC7291773; doi:10.3389/fpls.2020.00663)
Supplement: TABLE S2 — P-value data for Figures 1B–D, 4B–D. [file Table_2.pdf]

Supplementary Table 2. P-value data for Figure 1b/c/d and Figure 4b/c/d.

Plant	Chlorophyll content	Nitrate content	*ORE1* transcript
**Arabidopsis**	0.584535	2.61E-06	0.008142
**Pak Choi**	0.154849	4.37E-05	7.00406E-05
**Choy Sum**	0.385415	1.65E-06	0.000197
