# Supplementary material for: Early Diagnosis and Management of Nitrogen Deficiency in Plants Utilizing Raman Spectroscopy
Source: Front Plant Sci. 2020 Jun 5;11:663. doi: 10.3389/fpls.2020.00663 (PMC7291773; doi:10.3389/fpls.2020.00663)
Supplement: TABLE S3 — P-value data for Figure 2D. [file Table_3.pdf]

Supplementary Table3. P-value data for Figure 2d.

Plant	Nitrogen Deficiency	Phosphate deficiency	Potassium deficiency
**Arabidopsis**	0.005442373	0.406504012	0.875131679
