# Supplementary material for: Early Diagnosis and Management of Nitrogen Deficiency in Plants Utilizing Raman Spectroscopy
Source: Front Plant Sci. 2020 Jun 5;11:663. doi: 10.3389/fpls.2020.00663 (PMC7291773; doi:10.3389/fpls.2020.00663)
Supplement: TABLE S4 — P-value data for Figures 3B–D. ∗Nitrate peak indicated Raman spectrum at 1046 cm–1 and normalized with cartenoids peak (1520 cm–1). P value of nutrient-deprived growth (−N, −P, and −K, respectively) relative to Full medium as obtained from student’s t-test analysis (n=14–16). [file Table_4.pdf]

Supplementary Table 4. P-value data for Figure 3b, c and d.

Arabidopsis (Col-0 *and nrt2.1-2*)	Chlorophyll content	Nitrate content	*ORE1* transcript
**+N medium**	0.873644	1.95271E-06	0.001194
**-N medium**	0.444163	0.000848	0.038968
