# Supplementary material for: Early Diagnosis and Management of Nitrogen Deficiency in Plants Utilizing Raman Spectroscopy
Source: Front Plant Sci. 2020 Jun 5;11:663. doi: 10.3389/fpls.2020.00663 (PMC7291773; doi:10.3389/fpls.2020.00663)
Supplement: TABLE S5 — P-value data for intensity in Figures 3F, 4F. [file Table_5.pdf]

Supplementary Table 5. P-value data for intensity in Figure 3f and 4f.

Plants	Nitrate peak*
**Arabidopsis (Col-0 and *nrt2.1-2*) (+N)**	8.30134E-06
**Arabidopsis (Col-0 and *nrt2.1-2*) (-N)**	0.008678788
**Pak Choi (-N)**	3.43089E-05
**Choy Sum (-N)**	0.001104344

*Nitrate peak indicated Raman spectrum at 1046 cm^{-1} and normalized with carotenoids peak (1520 cm^{-1}). Table above lists P values of nutrient-deprived growth medium (-N, -P and –K, respectively) relative to Full medium as obtained from Student’s t-test analysis (n=14-16)
