# Supplementary material for: Early Diagnosis and Management of Nitrogen Deficiency in Plants Utilizing Raman Spectroscopy
Source: Front Plant Sci. 2020 Jun 5;11:663. doi: 10.3389/fpls.2020.00663 (PMC7291773; doi:10.3389/fpls.2020.00663)
Supplement: TABLE S6 — P-value data for Figures 5C,D. [file Table_6.pdf]

Supplementary Table 6. P-value data for Figure 5c and d.

**Arabidopsis**	**Nitrate content**	***ORE1* transcript**
+N and -N (0 d)	1.4E-08	0.007870944
+N and R (1 d)	1.15E-11	0.028305343
+N and R (2 d)	7.71E-09	0.896780143
+N and R (4 d)	0.002985	0.118211177
