# Supplementary material for: Early Diagnosis and Management of Nitrogen Deficiency in Plants Utilizing Raman Spectroscopy
Source: Front Plant Sci. 2020 Jun 5;11:663. doi: 10.3389/fpls.2020.00663 (PMC7291773; doi:10.3389/fpls.2020.00663)
Supplement: TABLE S7 — P-value data for Figure 7. [file Table_7.pdf]

Supplementary Table 7. P-value data for Figure 7.

Vegetables		Nitrate content	*ORE1* transcript
Pak Choi	+N(0)and R(0)	0.000761	0.0107838
	+N(1)and R(1)	0.037481	0.0641005
	+N(3)and R(3)	0.508053	0.7168413
Choy Sum	+N(0)and R(0)	0.000271	0.0085814
	+N(1)and R(1)	0.001392	0.0046007
	+N(3)and R(3)	0.071868	0.9882439
